# Multiple Horizontal Mini-chromosome Transfers Drive Genome Evolution of Clonal Blast Fungus Lineages

**DOI:** 10.1093/molbev/msae164

**Published:** 2024-08-06

**Authors:** Ana Cristina Barragan, Sergio M Latorre, Angus Malmgren, Adeline Harant, Joe Win, Yu Sugihara, Hernán A Burbano, Sophien Kamoun, Thorsten Langner

**Affiliations:** The Sainsbury Laboratory, University of East Anglia, Norwich Research Park, Norwich, UK; Department of Genetics, Evolution and Environment, Centre for Life's Origins and Evolution, University College London, London, UK; The Sainsbury Laboratory, University of East Anglia, Norwich Research Park, Norwich, UK; The Sainsbury Laboratory, University of East Anglia, Norwich Research Park, Norwich, UK; The Sainsbury Laboratory, University of East Anglia, Norwich Research Park, Norwich, UK; The Sainsbury Laboratory, University of East Anglia, Norwich Research Park, Norwich, UK; Department of Genetics, Evolution and Environment, Centre for Life's Origins and Evolution, University College London, London, UK; The Sainsbury Laboratory, University of East Anglia, Norwich Research Park, Norwich, UK; The Sainsbury Laboratory, University of East Anglia, Norwich Research Park, Norwich, UK

**Keywords:** clonal blast fungus lineages, crop disease pandemics, horizontal mini-chromosome transfer, wild hosts, genetic reservoirs

## Abstract

Crop disease pandemics are often driven by asexually reproducing clonal lineages of plant pathogens that reproduce asexually. How these clonal pathogens continuously adapt to their hosts despite harboring limited genetic variation, and in absence of sexual recombination remains elusive. Here, we reveal multiple instances of horizontal chromosome transfer within pandemic clonal lineages of the blast fungus *Magnaporthe* (Syn. *Pyricularia*) *oryzae*. We identified a horizontally transferred 1.2Mb accessory mini-chromosome which is remarkably conserved between *M. oryzae* isolates from both the rice blast fungus lineage and the lineage infecting Indian goosegrass (*Eleusine indica*), a wild grass that often grows in the proximity of cultivated cereal crops. Furthermore, we show that this mini-chromosome was horizontally acquired by clonal rice blast isolates through at least nine distinct transfer events over the past three centuries. These findings establish horizontal mini-chromosome transfer as a mechanism facilitating genetic exchange among different host-associated blast fungus lineages. We propose that blast fungus populations infecting wild grasses act as genetic reservoirs that drive genome evolution of pandemic clonal lineages that afflict cereal crops.

## Introduction

Coevolutionary dynamics between plants and their pathogens date back millions of years and act as a central force in shaping both sets of genomes (Barragan and Weigel 2021). In such antagonistically interacting organisms, a cycle of adaptation and counter-adaptation must occur to avoid extinction (Van Valen 1973). This evolution relies not only on the acquisition of novel mutations but also on the preservation of long-standing genetic variation; together, these components provide the genetic foundations upon which selective pressures act (Nei 2007; Barrett and Schluter 2008). In eukaryotes, one of the major sources of genetic variation is recombination through sexual mating, yet many organisms, including fungal plant pathogens, preferentially reproduce asexually (Barrett 2010; Möller and Stukenbrock 2017). The absence of sexual recombination necessitates alternative mechanisms for generating genetic variability, including mutations, genomic rearrangements, transposon insertion, and gene duplication or loss (Seidl and Thomma 2014; Oggenfuss et al. 2023). However, these processes rely primarily on pre-existing genetic variation, and without the introduction of new genetic material, the adaptive potential of an asexual population is constrained. How clonal plant pathogens adapt to their hosts and avoid extinction despite harboring limited genetic variation is an important research question with practical implications, as clonal lineages of plant pathogens often drive disease pandemics in crops (Drenth et al. 2019).

One mechanism for acquiring genetic variation which does not require sexual mating, is horizontal gene transfer (HGT). This process, consisting of the transmission of genetic material from a donor to a recipient organism within the same generation, is considered a major force in preventing extinction in asexual organisms (Takeuchi et al. 2014). In prokaryotes, HGT is well-established as a source of genetic diversity, occurring through known mechanisms such as conjugation, transformation or transduction (Sun 2018). The prevalence of HGT in eukaryotes has also become more apparent in recent years (Gabaldón 2020), particularly within the fungal kingdom—one of the most extensively studied eukaryotic lineage (Fitzpatrick 2012; Mohanta and Bae 2015; Sahu et al. 2023). In fungi, parasexuality, a mechanism enabling chromosome reassortment independent of sexual reproduction (Nieuwenhuis and James 2016), is a plausible avenue for HGT.

Fungal genes acquired by HGT are often part of the nonessential accessory genome which is variable between individuals of the same species and contrasts to the core genome, which contains genes essential to housekeeping functions (McCarthy and Fitzpatrick 2019). This is in line with the “two-speed” genome model observed in some filamentous plant pathogens (fungi and oomycetes), where indispensable genomic regions are under higher evolutionary constraints and may appear as slow-evolving, while variable genomic regions are under more relaxed constraints or positive selection, and can appear as rapidly evolving (Dong et al. 2015). Rapidly evolving or dynamic genome compartments are characterized by the presence of virulence genes, high sequence diversification, presence/absence variation, structural changes, and segmental duplications (Torres et al. 2020; Huang et al. 2023). An extreme form of structural variation are mini-chromosomes (mChr), also referred to as supernumerary, accessory, or B chromosomes, which exist in addition to core chromosomes and have been found in ∼15% of eukaryotic species (Covert 1998). While mChr emergence has been associated with genomic rearrangements at repeat- and effector-rich subtelomeric ends of core chromosomes (Bertazzoni et al. 2018; Peng et al. 2019; Langner et al. 2021; van Westerhoven et al. 2023), the exact molecular mechanism of how mChr emerge remain an area of ongoing investigation. By being physically unlinked from core chromosomes, mChr can diversify rapidly and could serve as a cradle for adaptive evolution without compromising genomic integrity (Croll and McDonald 2012).

The adaptive role of mChr in plant pathogenic fungi is underpinned by their correlation to virulence in various pathogen–host systems (Miao et al. 1991; Kistler 1996; Han et al. 2001; Akagi et al. 2009; Ma et al. 2010; Chuma et al. 2011; Balesdent et al. 2013; van Dam et al. 2017; Habig et al. 2017; Bhadauria et al. 2019; Henry et al. 2021; Asuke et al. 2023). In addition, variation in virulence has been partly attributed to the horizontal transfer of mChr (Mehrabi et al. 2011). This is exemplified in the case of *Fusarium oxysporum*, where the horizontal acquisition of a mChr in laboratory settings transformed a nonpathogenic strain into a virulent pathogen (Ma et al. 2010). Similarly, in the insect pathogen *Metarhizium robertsii,* strains with a horizontally acquired mChr were more virulent compared to those without this mChr (Habig et al. 2023).

A notorious plant pathogenic fungus where asexually reproducing clonal lineages underlie crop pandemics, is the blast fungus *Magnaporthe oryzae* (Syn. *Pyricularia oryzae*) (Latorre et al. 2020, 2023). The blast fungus is one of the most devastating plant pathogens worldwide and is the causal agent of blast disease in dozens of wild and cultivated grasses (Islam et al. 2023). As a species*, M. oryzae* is differentiated into genetic lineages that tend to be host-associated, with occasional gene flow observed between certain lineages (Couch et al. 2005; Gladieux et al. 2018a). To date, three globally prevalent clonal lineages affecting rice and one affecting wheat have been described as the underlying cause of persistent blast pandemics in agroecosystems (Latorre et al. 2020, 2023). Sexual reproduction in the highly destructive rice blast fungus lineage, which affects rice crops worldwide, is primarily confined to its center of origin in South Asia (Saleh et al. 2012; Thierry et al. 2022), with the remainder of the populations reproducing mostly asexually. These clonal rice blast fungus lineages remain genetically isolated, with no gene flow detected from other *M. oryzae* lineages so far (Gladieux et al. 2018a). Despite their restricted genetic diversity, clonal *M. oryzae* lineages readily evolve to counteract host defenses, posing a challenge to the development of durable blast-resistant crop varieties (Younas et al. 2023). The mechanism that allows clonal blast fungus populations to adapt to new host germplasm, despite an apparent lack of avenues for genetic innovation, remains elusive.

Structural variation in both mChr and core chromosomes contribute to genomic diversity in the blast fungus (Talbot et al. 1993; Orbach et al. 1996). Recent genomic analysis of a wheat blast fungus isolate revealed multi-megabase insertions from a related species, suggesting HGT (Kobayashi et al. 2023). In addition, the postulated horizontal transfer of the effector gene AVR-Pita2 among related species to the blast fungus substantiates the hypothesis that HGT is occurring (Chuma et al. 2011). While these instances highlight HGT as a possible driver of genetic variation in the blast fungus, the exact mechanisms facilitating HGT remain unclear. In addition to gene transfer, mChr have been associated with virulence gene reshuffling and recombination with core chromosomes (Kusaba et al. 2014; Peng et al. 2019; Langner et al. 2021; Asuke et al. 2023; Gyawali et al. 2023), indicating that horizontal mChr transfer could be instrumental in driving genomic innovation.

In this study, we provide evidence that multiple horizontal mChr transfer events involving clonal lineages of the rice blast fungus *M. oryzae* have occurred under field conditions. We identified a 1.2Mb accessory mini-chromosome, mChrA, which is remarkably conserved across *M. oryzae* isolates from lineages infecting the wild host species, Indian goosegrass (*Eleusine indica*), and rice. We show that mChrA was acquired by clonal rice blast fungus lineages through at least nine independent horizontal transfer events over the past three centuries. This establishes horizontal mChr transfer as a naturally-occurring genetic exchange mechanism among different host-associated blast fungus lineages. Our findings lead us to propose that blast fungus lineages infecting wild grasses serve as genetic reservoirs, driving genome evolution of pandemic asexual clonal lineages that afflict crops.

## Results

### Clonal Rice Blast Fungus Isolates Display Variable mChr Content

**Fig. 1.**
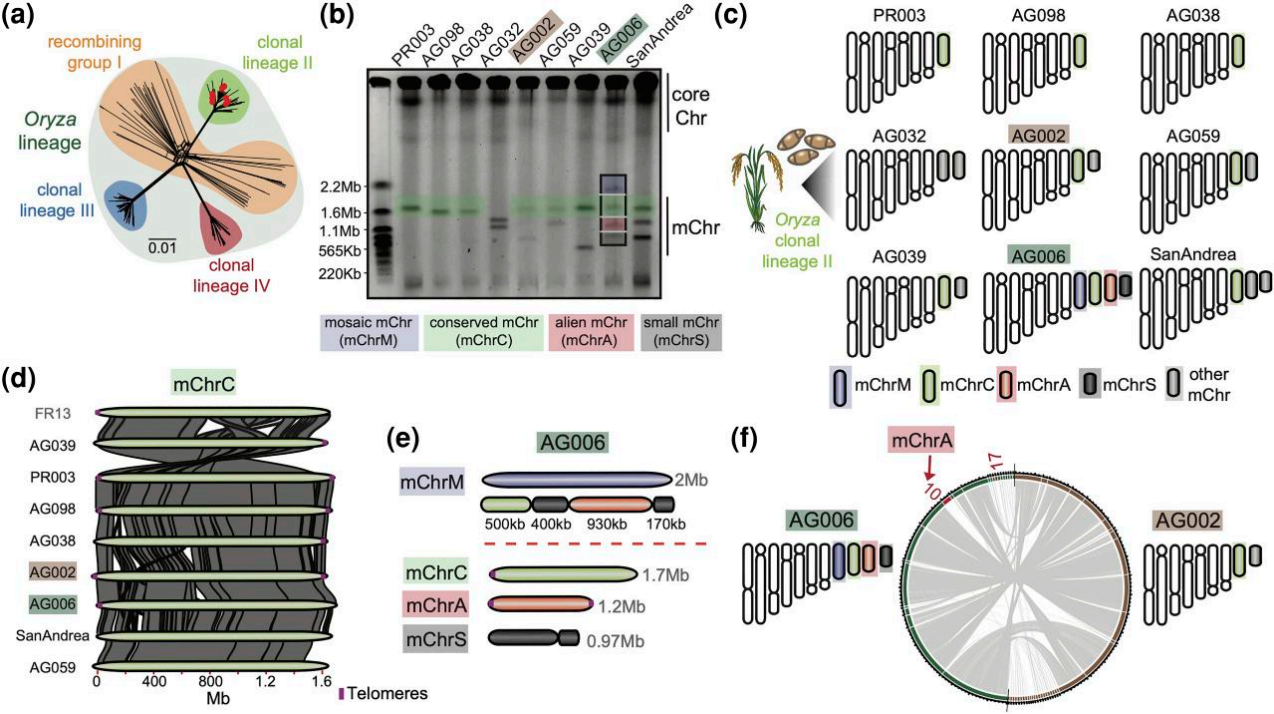
Clonal rice blast fungus isolates display variable mChr content. a) Genome-wide SNP-based NeighborNet analysis confirms the nine rice blast fungus isolates (red dots) belong to clonal lineage II (green) (Latorre et al. 2020). b**)** CHEF gel karyotyping reveals variable mChr content. A conserved 1.7Mb mChr (mChrC, green) is found in eight of nine isolates. A 2Mb mChr (mChrM, blue), present in isolate AG006, is a mosaic composed of fragments from three other mChr (mChrC, mChrA, and mChrS; see panel e) from the same isolate. A third 1.2Mb mChr (mChrA, red) found in AG006, is absent from the genomes of the other isolates (see panels E and F). c) Schematic karyotype of Italian isolates. Core chromosomes are shown in white. mChr studied in detail are highlighted in colors, while the rest are in gray. d**)** mChrC exhibits high synteny across isolates and is also found in isolate FR13 (Langner et al. 2021). Telomeric sequences are indicated by a vertical line (purple). e**)** Inferred mChrM sequence composition. f**)** Whole-genome alignment between AG006 (green) and AG002 (brown). mChrA (AG006_Contig10) and AG006_Contig17 (in red) are absent from AG002.

We have previously shown that genetically diverse *M. oryzae* isolates exhibit variable mChr content (Langner et al. 2021). Here, we set out to analyze the extent to which mChr variation contributes to genomic diversity in a set of genetically related isolates belonging to a single clonal lineage. To this end, we selected nine rice blast fungus isolates collected from Italy and generated chromosome-level whole-genome assemblies using short- and long-read sequencing technologies (Win et al. 2020) (supplementary fig. S1a, Supplementary Material online and supplementary table S1, Supplementary Material online). Using genome-wide single-nucleotide polymorphism (SNP) data we confirmed that the nine isolates belong to a single clonal lineage (clonal lineage II), which is predominant in Europe, and carry the mating type MAT1-2 (Latorre et al. 2020; Latorre et al. 2022b; Thierry et al. 2022) (Fig. 1a, supplementary S1b, Supplementary Material online and supplementary table S2, Supplementary Material online).

To determine the karyotype of the selected isolates, we performed contour-clamped homogeneous electric field (CHEF)-based electrophoresis. This revealed variable numbers and sizes of mChr, with each isolate exhibiting one to four mChr, each varying from 0.5 to 2Mb in size (Fig. 1b and 1c). To genetically characterize individual mChr, we performed mini-chromosome isolation sequencing (MCIS) on all eighteen mChr found across the nine isolates (Langner et al. 2019, 2021). Reads obtained from each individual mChr were mapped back to the corresponding reference assembly of their originating isolate, leading to the identification of mChr contigs (supplementary fig. S2, Supplementary Material online, supplementary S3, Supplementary Material online, supplementary tables S3 and S4, Supplementary Material online).

Next, we compared mChr contigs across the studied clonal isolates. Reciprocal sequence homology searches revealed the presence of a conserved 1.7Mb mChr (mChrC) in eight out of the nine isolates. mChrC corresponds to a previously identified mChr found in the rice blast fungus isolate FR13, which also belongs to clonal lineage II (Langner et al. 2021). We aligned mChrC contigs and confirmed high synteny across isolates (Fig. 1d). To obtain an overview of how common mChrC is in the global *M. oryzae* population, we examined the presence of this sequence across 413 *M. oryzae* and *Magnaporthe grisea* isolates (supplementary table S5, Supplementary Material online). We performed short-read mapping to the AG006 genome, known from karyotyping to possess the highest mChr diversity. Subsequent breadth of coverage calculations (see Methods) revealed that mChrC is particularly conserved among rice blast fungus isolates, especially those belonging to clonal lineage II (supplementary fig. S4a-c, Supplementary Material online and supplementary table S6, Supplementary Material online).

*M. oryzae* isolate AG006 stood out within the examined set of isolates as it contained three additional mChr named mChrS, mChrA, and mChrM, in addition to mChrC. These mChr exhibited sizes ranging from 0.97 to 2Mb. Notably, the largest of these mChr, which we termed the mosaic mini-chromosome (mChrM), was composed of segments derived from the three smaller mChr, namely, mChrC, mChrA, and mChrS (Fig. 1b and c, 1e and supplementary S2, Supplementary Material online). The presence of this mosaic mChr reveals that recombination among mChr occurs and plays a role in generating novel genetic combinations.

Upon closer examination of mChrA, we found it exhibited low sequence similarity to the genomes of the other Italian isolates, as evidenced by sequence homology searches and sequence alignments (Fig. 1e and supplementary tables S7 and S8, Supplementary Material online). There were two exceptions to this, a duplicated fragment within mChrM (Fig. 1e), and a small 0.1Mb contig (AG006_Contig17) which aligned to a specific region of mChrA (AG006_Contig10) (supplementary fig. S3a, Supplementary Material online). The latter may have originated from a sequence duplication event or be an assembly artifact. mChrA displayed high MCIS coverage and canonical telomeric repeats at both ends, indicating it is linear and largely assembled into a single contig (supplementary fig. S5, Supplementary Material online). Finally, to reinforce these findings, we conducted a whole-genome alignment between AG006 and AG002, an isolate genetically highly similar to AG006 (supplementary fig. S1b, Supplementary Material online), confirming the absence of mChrA in AG002 (Fig. 1f).

Taken together, we found high mChr diversity in a collection of nine clonal rice blast fungus isolates. Remarkably, we identified a unique mChr, mChrA, which does not display sequence similarity to the other nine rice blast fungus isolates. This finding underscores the unique genetic variation present even among closely related blast fungus isolates.

### mChrA Sequences Present Across Multiple Host-associated Blast Fungus Lineages

**Fig. 2.**
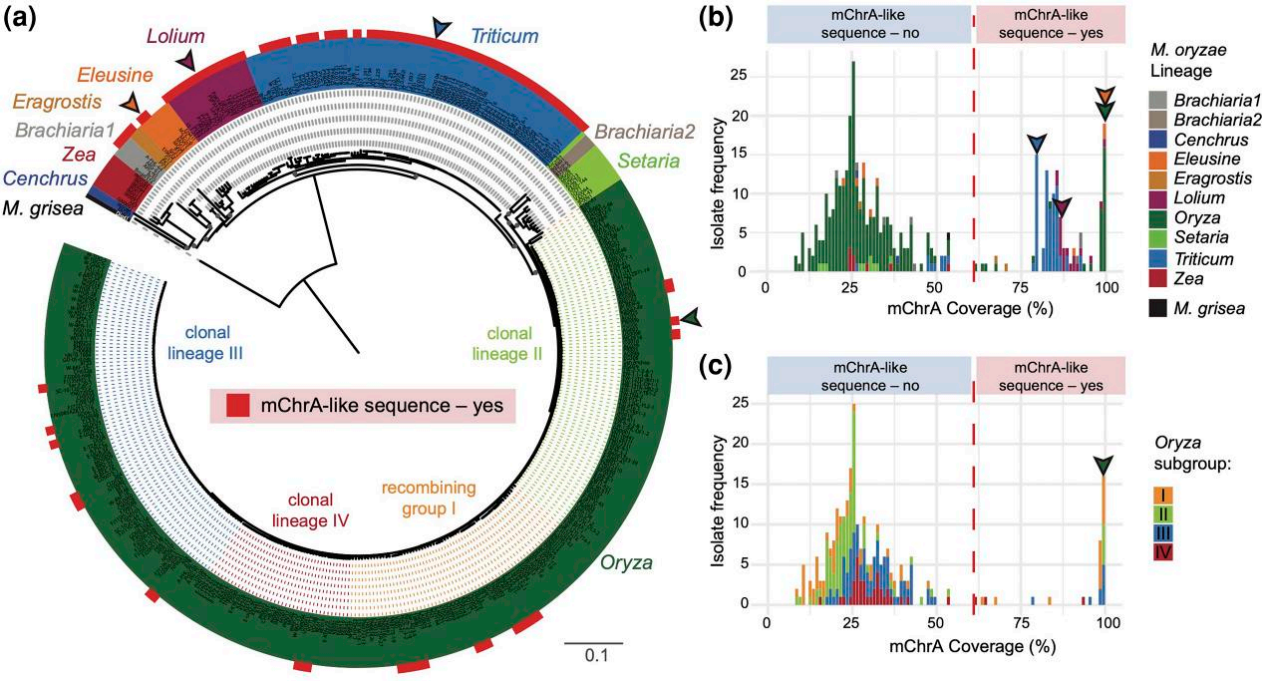
mChrA-like sequences are present across multiple host-associated blast fungus lineages. a) Genome-wide SNP-based NJ tree of 413 *M. oryzae* and *M. grisea* isolates. *M. oryzae* isolates are color-coded by lineage and *M. grisea* is in black. The 126 isolates defined as mChrA-like sequence carriers (supplementary table S9, Supplementary Material online) are highlighted by a red square and belong to six different *M. oryzae* lineages. Arrows indicate isolates with mChrA-related karyotyping information (Peng et al. 2019; Rahnama et al. 2020), see Fig. 4). Colors of dotted lines across the rice blast lineage represent different genetic subgroups (three clonal lineages and a recombining group) (Latorre et al. 2020). Scale bar represents nucleotide substitutions per position. b**)** Bimodal distribution of mChrA breadth of coverage across 413 *M. oryzae* and *M. grisea* isolates. The coverage cutoff (61%) for mChrA-like sequence presence or absence is indicated by the dotted red line. Arrows as in (a). **c)** mChrA breadth of coverage across 276 rice blast fungus isolates. Colors represent different genetic subgroups in the *Oryza* lineage. Arrows and coverage cutoff as in (b).

To determine the origin of mChrA, we assessed the presence of the mChrA sequence (AG006_Contig10) across a set of 413 *M. oryzae* isolates belonging to ten different host-associated lineages and to *M. grisea* (Fig. 2a and supplementary table S5, Supplementary Material online). For this purpose, we calculated the breadth of coverage for mChrA (AG006_Contig10) in each isolate, defined as the percentage of sequence covered by one or more reads from a particular isolate which mapped to the AG006 reference, and observed it followed a bimodal distribution (Fig. 2b). Model-based clustering established 126 isolates as carriers of mChrA-like sequences (supplementary fig. S6a-c, Supplementary Material online and supplementary table S9, Supplementary Material online, see Methods). These isolates belonged to six different host-associated *M. oryzae* lineages (Fig. 2a and b and supplementary S6d, Supplementary Material online). Notably, only 12% of rice blast fungus isolates (32 of 276) were identified as mChrA-like sequence carriers (Fig. 2a and b and supplementary table S9, Supplementary Material online). These rice blast fungus isolates were genetically diverse, belonging to all three clonal lineages and the recombining group (defined here as *Oryza* subgroups) (Fig. 2c and supplementary S6e, Supplementary Material online).

In contrast to rice blast fungus isolates, mChrA-like sequences were common across isolates belonging to the *Lolium* and *Triticum* lineages, with most isolates carrying 80% to 90% of the mChrA (Fig. 2b and supplementary S6d, Supplementary Material online). Previous karyotyping and sequencing efforts identified isolate B71 from the *Triticum* lineage and isolate LpKY97 from the *Lolium* lineage to carry mChr (Peng et al. 2019; Rahnama et al. 2020). Given that these isolates also carry a substantial portion of mChrA sequence (79% and 87% mapping to mChrA in AG006, respectively), it suggests that the mapped mChrA-like sequences may share a common ancestry with the mChr in these two isolates. However, the most striking sequence identity was observed in two isolates from the *Eleusine* blast fungus lineage, Br62 and B51. These exhibited mChrA coverage comparable to the rice blast fungus isolate AG006, suggesting high similarity in the mChrA-like sequences between these isolates (Fig. 2b and supplementary table S6, Supplementary Material online).

Next, to rule out the possibility of spontaneous loss of mChrA under laboratory conditions and its relation to the time period each isolate has been cultured in the laboratory, we examined the statistical relationship between the isolate collection year and mChrA presence/absence. The similar distributions of collection dates and mChrA presence/absence indicate that the year of collection does not influence our observations (Wilcoxon test *P*-value = 0.6877, supplementary fig. S7, Supplementary Material online). Furthermore, we conducted a logistic regression analysis to assess the probability of mChrA presence/absence based on the collection date. The regression coefficient was not statistically significant (*P*-value = 0.814), indicating that the collection date does not affect the presence/absence of the mChrA sequence.

Taken together, mChrA-like sequences were found in blast fungus isolates belonging to six different host-associated lineages, with members of the *Eleusine* and *Oryza* lineages carrying nearly identical mChrA sequences.

### Discordant Genetic Clustering Between the Core Genome and mChrA

**Fig. 3.**
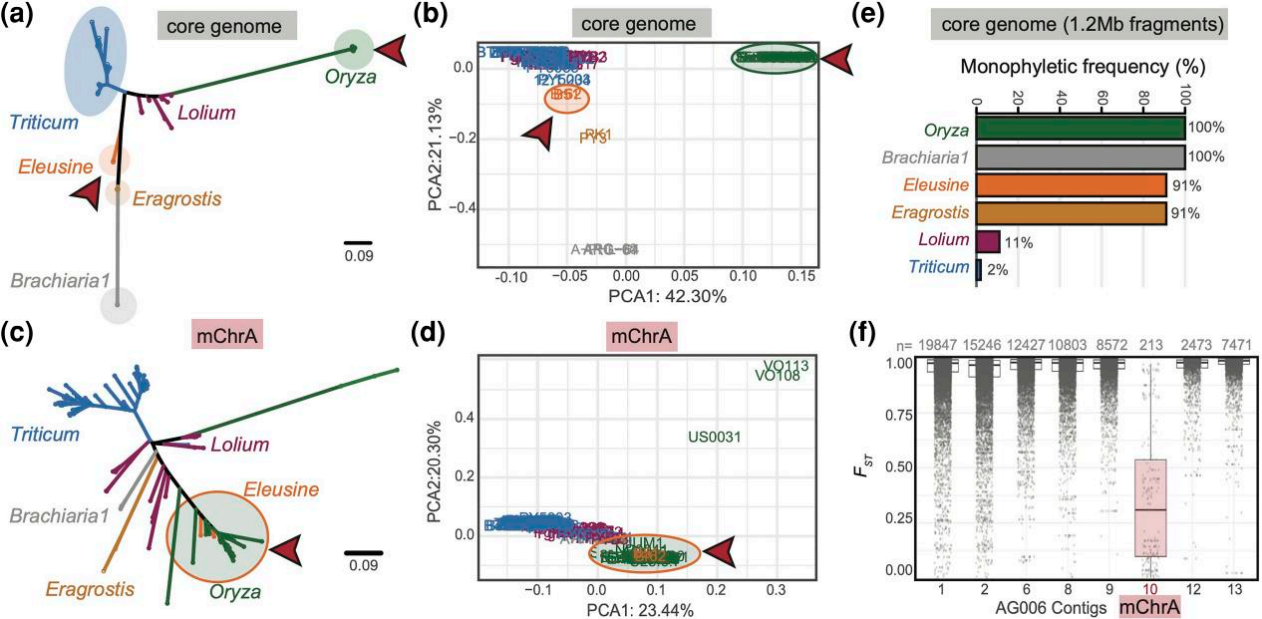
Discordant genetic clustering between the core genome and mChrA. a-d). SNP-based NJ trees (a and c) and Principal Component Analyses (PCA, b and d) of 126 *M. oryzae* isolates carrying the mChrA-like sequence. Discordance between core genome (a and b) and mChrA (c and d) genetic clustering is observed (highlighted by red arrows). Scale bar represents nucleotide substitutions per position. e**)** Percentage of tree topologies where a monophyletic relationship was observed for 100 randomly selected 1.2Mb core-chromosomal regions. In all instances the *Oryza* lineage was monophyletic, and in no instance did the *Eleusine* and *Oryza* blast fungus lineages cluster together. f) *F_ST_* between rice blast fungus isolates (n = 32) and isolates from the *Eleusine* lineage (Br62 and B51) both carrying mChrA-like sequences. Each dot (gray) indicates the weighted *F_ST_* per 5 kb window using a step size of 500 bp. The number of windows per contig are at the top of each box. Core chromosome contigs >2Mb and mChrA are shown.

Given the patchy distribution of mChrA-like sequences across isolates from different host-associated blast fungus lineages, we investigated the evolutionary relationships of their core genome and mChrA-like sequences. Using SNP-based phylogenies and principal component analyses (PCAs) (supplementary fig. S8, Supplementary Material online), we found a clear discordance between the core genome and mChrA (Fig. 3a-d). For the core genome, isolates are clustered by lineage, whereas for mChrA-like sequences, lineage-dependent clustering becomes less evident. Most strikingly, whereas the core genomes of isolates from the *Oryza* and *Eleusine* lineages form two distinct groups (Fig. 3a and b), these two fall within a single group for mChrA (Fig. 3c and d). This shows that the mChrA in isolates from these two lineages is highly similar, but their core genome is divergent. We note that for the mChrA clustering, three clonal rice blast fungus isolates did not group with other isolates from this lineage, based on SNP-based phylogenies and PCAs, possibly reflecting mChrA sequence dissimilarity.

To ascertain the robustness of the observed genetic clustering of mChrA-like sequences between members of the *Oryza* and *Eleusine M. oryzae* lineages, we generated phylogenies using 100 randomly selected genomic regions of the same size as mChrA (1.2Mb) across the core genome of all 126 isolates. In all instances, the *Oryza* lineage was monophyletic, and in zero instances did the *Oryza* and *Eleusine* lineages cluster together (Fig. 3e). This demonstrates that the clustering of members of the *Eleusine* and rice blast fungus lineages is highly unusual and limited to the mChrA sequence. To complement this analysis, we evaluated genetic differentiation between isolates belonging to the rice and *Eleusine* blast fungus lineages carrying mChrA-like sequences by calculating the fixation index (F*_ST_*) from genome-wide SNP data (Wright 1951). This analysis confirmed high levels of inter-lineage genetic differentiation in the core genome, but low differentiation levels for mChrA (Fig. 3e). Finally, ancestry estimation using ADMIXTURE (Alexander and Lange 2011) supported common ancestry among isolates from the *Oryza* and *Eleusine* lineages to present and limited to mChrA (supplementary fig. S9, Supplementary Material online). We conclude that mChrA shows discordant genetic clustering when compared to the core genome, which indicates contrasting evolutionary trajectories.

### *Eleusine* Isolate Br62 and *Oryza* Isolate AG006 Carry an Intact and Highly Syntenic mChrA

**Fig. 4.**
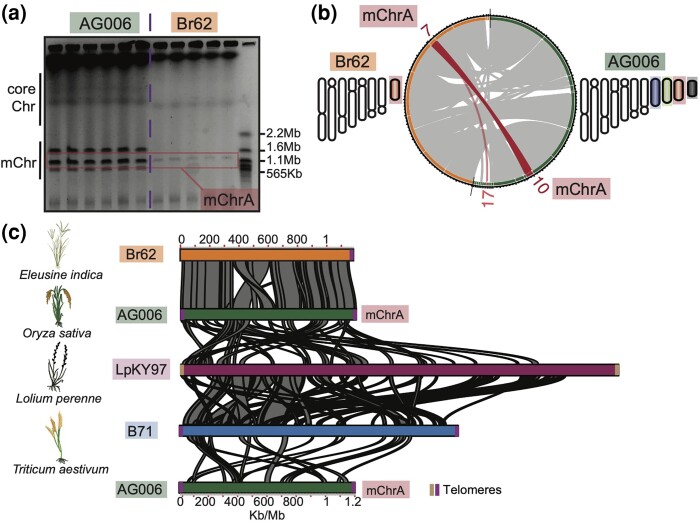
*Eleusine* isolate Br62 and *Oryza* isolate AG006 carry an intact and highly syntenic mChrA. a) CHEF-gel karyotyping of AG006 and Br62. Six gel lanes per isolate are shown representing a single biological replicate. Br62 carries a 1.2Mb mChr, the same size as mChrA in AG006 (in red). b**)** Whole-genome alignment of Br62 (orange) and AG006 (green). Br62_Contig07 aligns exclusively to mChrA (AG006_Contig10) and AG006_Contig17 (red). For both isolates a schematic karyotype is depicted. c**)** Alignment of mChrA in AG006 and Br62 reveal high synteny, except for a rearrangement in the central region. Alignments covering a fraction of mChrA are seen among mChrA in AG006 and the mChrA-like mChr1 in *Lolium* isolate LpKY97 (magenta) and the mChr in *Triticum* isolate B71 (blue). Telomeric sequences are indicated by vertical lines (purple/brown). The host plant of each isolate is shown on the left.

Following the identification of highly similar mChrA sequences in two isolates from the *Eleusine* blast fungus lineage, we aimed to determine whether these sequences originate from an intact mChr, or whether they are embedded within the core genome, as observed for mChrC segments in *M. oryzae* isolate 70–15 (Langner et al. 2021). To test this, we performed CHEF-gel-based karyotyping, and found that Br62 possesses a single mChr of the same size (1.2Mb) as mChrA in AG006 (Fig. 4a). We performed whole-genome sequencing of Br62 using both Illumina short reads and Nanopore long reads, followed by de novo whole-genome assembly (supplementary table S10, Supplementary Material online). Subsequent whole-genome alignment between AG006 and Br62 revealed that Br62_Contig07 corresponds to mChrA (AG006_Contig10) and AG006_Contig17 (Fig. 4b). Furthermore, the alignment of the mChrA in both isolates revealed a high level of synteny, with a single re-arrangement in the center of mChrA (Fig. 4c).

To independently validate these findings, we took advantage of a Br62 isolate that lost mChrA after subculturing, as determined by CHEF gel electrophoresis (supplementary fig. S10a, Supplementary Material online). To identify contigs that originate from the mChr, we sequenced the genome of the Br62 isolate that lacks the 1.2Mb mChr (referred to as Br62-) using Illumina short-reads and aligned the reads to the Br62 genome. We calculated mapping depth per contig in Br62 and Br62-. Depths were consistent in both isolates except for Contig07, here Br62- displayed a near-zero read depth, indicating this corresponded to mChrA (supplementary fig. S10b, Supplementary Material online). Additionally, Contig07 exhibited a high repeat content, a characteristic feature of mChr (supplementary fig. S10c, Supplementary Material online). Together these analyses confirm the presence of an intact mChrA in Br62. Intriguingly, subculturing not only resulted in the loss of mChrA in Br62 but also in the loss of the mosaic mChrM in AG006 (Fig. 4a), underlining the dynamic nature of mChr (Peng et al. 2019; Langner et al. 2021; Liu et al. 2022).

We next set out to determine whether the mChrA sequence is also found as an intact mChr in isolates belonging to other blast fungus lineages known to carry mChr (Peng et al. 2019; Rahnama et al. 2020), and which we identified as carriers of mChrA-like sequences (supplementary table S9, Supplementary Material online). We performed pairwise whole-genome alignments between AG006, isolate LpKY97 from the *Lolium* lineage, and isolate B71 from the *Triticum* lineage. Here, mChrA partially aligned to the mChr of both B71 and LpKY97, and to the end of chromosome 3 in B71, which was previously identified as a potential segmental duplication between the B71 mChr and core chromosomes (Peng et al. 2019; Liu et al. 2022; Gyawali et al. 2023) (Fig. 4c and supplementary fig. S11a-c, Supplementary Material online). The partial mChrA alignments are in accordance with our genetic clustering and breadth of coverage analyses, indicating that mChrA-like mChr are present in LpKY97 and B71, but these are structurally divergent from mChrA in AG006 and Br62 (supplementary table S6, Supplementary Material online). As a negative control, we aligned mChrA from AG006 to the conserved mChrC in the rice blast fungus isolate PR003 using the same parameters and no alignments were retrieved. We conclude that mChrA is present as an intact and highly syntenic mChr in the rice blast fungus isolate AG006 and in *Eleusine* blast fungus isolate Br62.

Having established that mChrA is present as an intact mChr in AG006 and Br62, we screened and compared the genetic composition of mChrA in these two isolates. The mChrA in AG006 and Br62 displayed highly similar characteristics clearly distinguishing them from the core chromosomes. Both mChrA displayed a lower density of predicted genes, a high repeat and a lower GC content (supplementary fig. S12a and b, Supplementary Material online, supplementary tables S11 and S12, Supplementary Material online). In addition, mChrA in AG006 carried eight putative secreted proteins, which are candidate virulence effectors, while mChrA in Br62 carried seven (supplementary fig. S12c and d, Supplementary Material online, supplementary tables S11 and S12, Supplementary Material online). For each mChrA, we predicted their protein content using InterProScan (Jones et al. 2014) (supplementary tables S13 and S14, Supplementary Material online), and extracted Gene Ontology (GO) terms for proteins we annotated with a known or predicted or function (supplementary tables S15 and S16, Supplementary Material online). We found that 43 out of 49 (88%) unique GO terms were shared in the mChrA of Br62 and AG006 (supplementary fig. S13, Supplementary Material online and supplementary table S17, Supplementary Material online). Collectively, the sequence composition and protein content of mChrA AG006 and Br62 is highly similar to each other and distinct to that of the core genome.

### Multiple Horizontal mChrA Transfers Occurred in Clonal Rice Blast Fungus Lineages

**Fig. 5.**
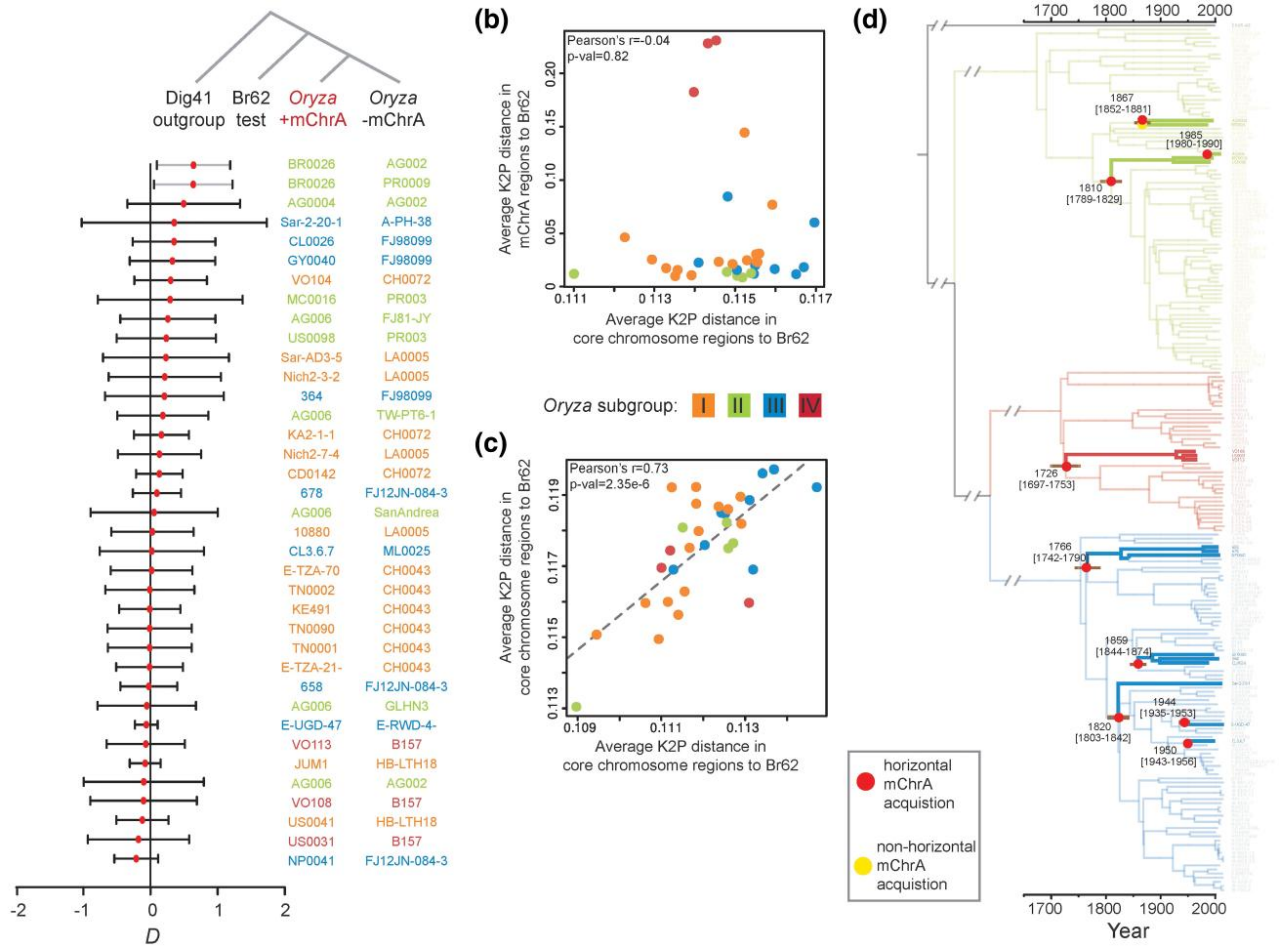
Multiple mChrA transfers occurred in clonal rice blast fungus lineages. a) *D*-statistics. Lines depict 99% confidence intervals and the red dot the estimated *D* value. Lines not encompassing *D* = 0 are gray and the rest black. Jack-knife blocks were 5Mb long. b**)** Average Kimura two-parameter (K2P) distances between homologous mChrA sequences from +mChrA isolates to Br62, and average distances of random core chromosome homologous sequences from +mChrA isolates to Br62. c**)** Average K2P genetic distances between two sets of homologous random core chromosome sequences from +mChrA isolates to Br62. d**)** Ancestral states of mChrA presence or absence along the clonal rice blast fungus phylogeny. Thick lines indicate mChrA is present. Branches are color-coded by lineage. The SA05-43 isolate from the *Setaria* blast fungus lineage was chosen as an outgroup. Branches with evidence for horizontal mChrA acquisition are indicated by a red diamond, the branch where there is evidence for sexual transfer is indicated by a yellow diamond. The brown bars in the nodes represent the 95% Highest Posterior Density.

To test if sexual mating or HGT can explain the presence of mChrA in the *Eleusine* and *Oryza* blast fungus lineages, we evaluated patterns of allele sharing through *D*-statistics (Green et al. 2010; Durand et al. 2011). After the mChrA sequence is removed from the genomes, we hypothesize that sexual mating results in a genome-wide introgression signal, leading to a *D*-statistic significantly different from zero, whereas HGT will not produce such a signal. Consequently, we first removed mChrA-like sequences, and then compared Br62 with the 32 rice blast fungus isolates carrying mChrA sequences, and 13 rice blast isolates not carrying this sequence (see Methods). For each comparison, rice blast fungus isolates belonging to the same *Oryza* subgroup were chosen. We selected *M. grisea* isolate Dig41 as an outgroup, which is divergent from both the rice and *Eleusine* blast fungus lineages. This resulted in the phylogenetic configuration: (Dig41, Br62; *Oryza* +mChrA, *Oryza* -mChrA). Under this configuration, a 99% confidence interval encompassing *D* = 0 indicates there is no genome-wide introgression signal, and favors the hypothesis of horizontal mChrA transfer. On the other hand, a 99% confidence interval not encompassing *D* = 0 signals genome-wide introgression, supporting the acquisition of mChrA through sexual mating. In all tested configurations except those involving isolate BR0026 (31 of 32 isolates), the 99% confidence interval encompassed *D* = 0, supporting the acquisition of mChrA by horizontal transfer (Fig. 5a and supplementary table S18, Supplementary Material online). As a control, we tested the configurations (Dig41, Br62; *Oryza* +mChrA, *Oryza* +mChrA) (supplementary fig. S14a, Supplementary Material online) and (Dig41, Br62; *Oryza* -mChrA, *Oryza* -mChrA) (supplementary fig. S14b, Supplementary Material online and supplementary table S18, Supplementary Material online). Here, the 99% confidence interval encompassed *D* = 0 in all tested configurations. To test the power of *D*-statistics in detecting ancestral introgression events, we carried out simulations under the assumption of a single pulse of introgression followed by multiple backcrosses (see Materials and Methods). Our results show that the detection power extends from hundreds to even thousands of generations, indicating that even rare ancestral introgression events can be detected (supplementary fig. S15, Supplementary Material online).

Having established that mChrA was likely horizontally acquired in the large majority of rice blast fungus isolates carrying this sequence (31 of 32 isolates), and given its patchy distribution across the rice blast fungus lineage, we sought to differentiate between a single horizontal ancestral mChrA acquisition followed by independent losses, and multiple independent horizontal mChrA acquisitions. To test this, we estimated the genetic distance between nonoverlapping and contiguous 100 kb windows of the mChrA sequence, present in the 32 *Oryza* isolates and Br62, and compared it to the genetic distance between nonoverlapping random core chromosome regions of the same size, in the same 32 *Oryza* isolates and Br62. A significant correlation between the two genetic distances indicates that both the mChrA and the core chromosomes have accumulated mutations in a correlated way. This would support a single ancestral mChrA acquisition by the *Oryza* lineage, followed by multiple mChrA losses, whereas a lack of correlation suggests independent mChrA acquisitions. By analyzing correlations between genetic distances relative to Br62 instead of comparing their magnitudes, our analysis is not confounded by changes in mutation rate or different strengths of purifying selection operating at the mChrA and core chromosome level. We did not find any correlation between the genetic distances of the mChrA region from +mChrA isolates to Br62, and the genetic distances of core chromosome regions from +mChrA isolates to Br62 (Fig. 5b). As a control, we compared genetic distances between two sets of random core chromosome regions from +mChrA isolates to Br62 and found a strong correlation (Fig. 5c). Together, these results favor the hypothesis that the observed mChrA distribution in the rice blast fungus lineage is the result of multiple independent mChrA acquisitions.

In addition, we reconstructed the ancestral states of mChrA presence or absence along the clonal rice blast fungus phylogeny and found evidence for nine independent horizontal mChrA acquisitions. Using a time-scaled phylogeny, we could time these events to have occurred within the past three centuries (Fig. 5d).

Finally, since mChr can be generated as the result of genomic rearrangements from core chromosomes (Langner et al. 2021), this allowed us to test the directionality of the horizontal mChrA transfer. We hypothesized that a lineage where a mChr originated will have greater similarity between its core chromosomes and that mChr compared to a different lineage that might receive it. Using this logic, we measured patterns of k-mer sharing between core chromosomes and mChrA in the *Eleusine* Br62 isolate and the rice blast fungus isolate AG006. We observed a higher number of kmer-sketches shared between Br62 core chromosomes and the Br62 mChrA (25,060/1,000,000) in contrast to AG006 core chromosomes and AG006 mChrA (3,903/1,000,000). Moreover, core chromosome regions from Br62 shared a higher number of kmer-sketches with the AG006 mChrA (14,802/1,000,000) than those shared between AG006 core chromosomes and Br62 mChrA (6,597/1,000,000) (supplementary table S19, Supplementary Material online). These results support a likely directionality of mChrA from the *Eleusine* into the rice blast fungus lineage.

In summary, we provide compelling evidence supporting the scenario of multiple horizontal mChrA transfers involving members of the *Eleusine* and *Oryza* blast fungus lineages. A minimum of nine independent mChrA acquisitions and multiple independent losses occurred across clonal rice blast fungus lineages over the past three centuries.

## Discussion

Crop disease pandemics are frequently caused by clonal lineages of plant pathogens that reproduce asexually. The mechanisms enabling these clonal pathogens to adapt to their hosts, despite their limited genetic variation, remain an area of active research. In our study, we demonstrate that mChr serve as a source of genetic variation for asexual clonal pathogens. We observed horizontal mChr transfer occurred in field isolates belonging to clonal populations of the rice blast fungus *M. oryzae*. Our findings demonstrate horizontal acquisition of a 1.2Mb supernumerary mChr by clonal rice blast isolates from a genetically distinct lineage infecting *Eleusine indica*, a wild grass species. We identified a minimum of nine independent horizontal mChr acquisitions over the past three centuries. This establishes horizontal mChr transfer as a process facilitating genetic exchange between host-associated blast fungus lineages in the field. We propose that blast fungus populations infecting wild grasses serve as genetic reservoirs for clonal populations infecting cultivated crops. Horizontal acquisition of mChr by clonal blast fungus isolates appears to increase their genetic diversity, driving genome evolution and potentially aiding in its adaptability.

The genetic mechanisms underlying horizontal mChr transfer in clonal fungus isolates are intriguing. Under laboratory conditions, horizontal transfer of mChr between fungal isolates has been facilitated through methods such as protoplast fusion (Akagi et al. 2009) or co-culturing (Masel et al. 1996; He et al. 1998; Ma et al. 2010; Vlaardingerbroek et al. 2016; van Dam et al. 2017). Underlying these mChr transfers is parasexual recombination (Soanes and Richards 2014; Vlaardingerbroek et al. 2016). Here, cells from different individuals fuse via anastomosis and form heterokaryons (Roca et al. 2003, 2005; Ishikawa et al. 2010; Vangalis et al. 2021). These heterokaryons can become unstable diploid cells, undergoing chromosome reassortment during mitosis (Strom and Bushley 2016). In the case of *Magnaporthe* spp. heterokaryon formation has been achieved under laboratory conditions by co-culturing isolates, indicating that parasexuality is possible (Crawford et al. 1986). This mechanism has been suggested as a source of genetic variation in the rice blast fungus, potentially occurring under field conditions (Zeigler et al. 1997; Noguchi et al. 2006; Tsujimoto 2011; Monsur and Kusaba 2018). Here, we found robust evidence that horizontal mChr transfer occurs under field conditions, a process probably parasexual in nature.

Parasexuality offers fungi an alternative route to enhancing genetic diversity, while maintaining relative genomic stability and avoiding the complexities of sexual reproduction, including premating barriers like reproductive timing and postmating issues such as hybrid incompatibilities (Roper et al. 2011; Stukenbrock 2013). Recently, it was proposed that chromosome reassortment during parasexual recombination may not be entirely random (Habig et al. 2023). Here, it was suggested that some mChr are preferentially transferred or tend to resist loss or decay compared to others, resembling the behavior of selfish genetic elements (Ahmad and Martins 2019). This phenomenon could be attributed to distinct chromatin conformations of the mChr. Future research will investigate whether mChrA carries chromatin remodeling elements that could enable its horizontal transfer or shield it from degradation, potentially elucidating the relatively frequent horizontal transfer events observed across the rice blast fungus lineage. Moreover, to better understand the impact of horizontal mChr transfer on *M. oryzae* evolution, it will be crucial to study how frequent and diverse these events are in field populations.Not all instances of inter-lineage transfer events of the mChrA sequence seem to be the product of parasexually-mediated horizontal transfer. Hybridization through sexual mating is a major player shaping the evolution of fungal plant pathogens, bringing forth a myriad of novel genetic combinations for selection pressures to act on (Stukenbrock 2016). In *M. oryzae*, there is evidence of sexual mating occurring both within and between specific host-associated blast fungus lineages, occasionally facilitating host jumps (Gladieux et al. 2018b). In our study, one clonal rice blast fungus isolate, BR0026, exhibited genome-wide introgression signals with *Eleusine* isolate Br62 (Fig. 5a and supplementary table S18, Supplementary Material online). One plausible hypothesis is that the introgression signals observed in BR0026 may reflect ancient sexual reproduction events involving an isolate from the *Eleusine* lineage. Given that these two isolates were collected in South America, it is possible that sympatry in this region led to sexual reproduction between members of the rice and *Eleusine* lineages.

In addition to isolates belonging to the *Eleusine* and *Oryza* blast fungus lineages, isolates belonging to the *Triticum* and *Lolium* lineages also carry mChrA-like sequences. It remains to be determined whether these were obtained via horizontal transfer or sexual reproduction. The absence of genetic discordance between the core chromosomes and mChrA in these isolates supports sexual reproduction (Fig. 4a-d). In addition, substantial admixture has been observed among members of the *Triticum* and *Lolium* lineages (Gladieux et al. 2018b), suggesting that sexual reproduction may be the route through which mChrA-like sequences were acquired.

Our study on the prevalence of horizontal gene exchange within local populations underscores the importance of accounting for ecological factors, especially in fungi that tend to specialize on specific hosts. Although our global comprehension of blast fungus populations has expanded, the detailed study of local populations, particularly those that include isolates from both wild and cultivated hosts, remain scarce (Cruz and Valent 2017; Barragan et al. 2022). One question to address will be how horizontal gene exchange through parasexuality is enabled in natural environments. In the case of the blast fungus, one factor offering an avenue for genetic interchange may be the absence of strict host-specialization (Gladieux et al. 2018a). Laboratory studies have shown that hosts like barley and common millet are susceptible to genetically distinct blast fungus lineages (Kato et al. 2000; Hyon et al. 2012; Chung et al. 2020). In the field, some cases of cross-infection have been reported, but the extent to which these occur in local populations is unknown (Gladieux et al. 2018a). Such susceptible hosts could serve as hubs for genetic exchanges, potentially contributing to horizontal mChr transfers between isolates from different lineages. Moreover, being a facultative biotroph, the blast fungus possesses the ability to thrive on both living plants and saprophytically on decaying plant matter. This broadens the window for possible genetic interactions, as the pathogen does not require synchronous growth within the same living hosts for this to occur. Understanding gene flow within local blast fungus populations through the study of HGT and other mechanisms, is vital for developing effective disease management strategies. For example, identifying frequent horizontal gene exchange between isolates infecting specific hosts could lead to targeted measures such as strategic weeding or focused fungicide application.

One persistent challenge in pinpointing elements of the accessory genome, such as mChr, has been the biases arising from aligning sequencing reads to a single reference genome. In past comparative genomic approaches, mChrA went unnoticed, as we aligned isolates carrying this sequence to the MG08 reference genome from isolate 70–15, which lacks the mChrA sequence. Leveraging methods such as pan-genomes, which are gaining traction across the fungal kingdom (Badet and Croll 2020) or de novo assemblies using short read data (Potgieter et al. 2020), coupled with reference-independent genetic clustering approaches like k-mer (Zielezinski et al. 2017; Aylward et al. 2023) and read-based (Dylus et al. 2024) techniques, promises more accurate identification of mChr and of horizontally introgressed regions. The latter could be detected by first identifying mChr and other accessory genomic elements, and then comparing them with the core genome. Studies of this nature have recently detected cases of horizontal introgression in other fungal pathogens (Moolhuijzen et al. 2022; Petersen et al. 2023). In addition to these approaches, the integration of artificial intelligence to distinguish between core chromosomes and mChr using short-read sequencing data presents a timely and innovative approach (Gyawali et al. 2023). The successful implementation of such methodologies will not only facilitate the large-scale identification of candidate mChr regions across isolates, but also help establish whether these regions are preferentially involved in horizontal transfer events.

The horizontal transfer of mChrA from a blast fungus lineage that infects the wild grass *Eleusine indica* to clonal rice blast fungus lineages underscores the intricate ecological interactions involved. Wild grasses can act as potential genetic reservoirs, echoing the dynamics observed in zoonotic diseases where pathogens jump between wild animals and humans (Rahman et al. 2020). This analogy between the plant and animal realms highlights the significance of wild species as reservoirs of pathogens and suggests the possibility of genetic transfers. However, surveys on blast disease often focus on cultivated crops, neglecting wild hosts (Barragan et al. 2022). Therefore, enhanced awareness and surveillance of gene flow dynamics in local blast fungus populations are necessary. This should include investigations into the role of wild grasses as genetic conduits, similar to the concept of zoonoses. Such understanding is crucial for the early identification and prevention of genetic transfers that could initiate new disease outbreaks or intensify existing ones.

## Conclusion

**Fig. 6.**
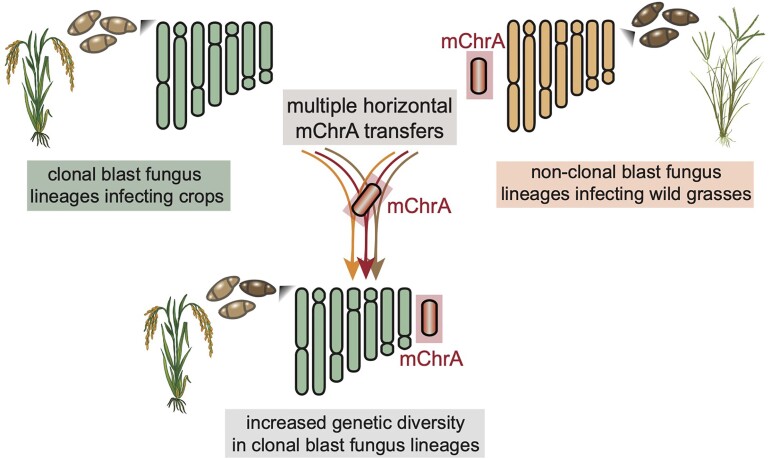
Model. Horizontal mini-chromosome transfers from blast fungus lineages infecting wild grasses drive genome evolution of clonal lineages infecting crops. The recurrent acquisition of mChrA from wild grass-infecting blast fungus lineages by clonal rice blast fungus lineages enhances their evolutionary adaptability and capacity to respond to changing environments and hosts. The coexistence of infected crops and wild hosts facilitates this genetic exchange, posing a challenge to the management of crop disease pandemics.

Clonal isolates of the blast fungus are a significant agricultural concern due to their central role in causing crop disease pandemics. The key to tackling this issue is to understand how genetically uniform populations adapt to novel hosts. Our research has revealed that supernumerary mChr undergo horizontal transfer in natural field conditions. Notably, we found that mChrA has been transferred horizontally on multiple independent occasions involving isolates from a lineage of blast fungus affecting a wild grass and clonal lineages infecting rice. This finding sheds light on the role of horizontal mChr transfer in driving the genome evolution of clonal blast fungus populations, potentially aiding in host adaptation. Isolates originating from wild grasses may act as reservoirs of genetic diversity (Fig. 6). These insights underscore the importance of disease surveillance that encompasses both agricultural crops and adjacent wild grass species.

## Materials and Methods

### Blast Fungus Growth Conditions

Blast fungus isolates were grown from filter paper stocks by placing these on complete medium (CM) for 7 to 14 d in a growth chamber at 24°C with a 12-h light period to induce growth of mycelium and sporulation. For liquid cultures, 8 to 10 small blocks of mycelium (ca. 0.5 cm × 0.5 cm) were cut out of the edge of fully grown colonies with a sterile spatula, transferred into 150 ml of liquid CM medium in a 250-ml Erlenmeyer flask and incubated on a rotary shaker at 120× *g* and 24°C for 2 to 3 d.

### Visualization of Worldwide Blast Fungus Distribution

Maps showing the geographical locations of the studied blast fungus isolates were plotted with the R-package ggmap (v3.0) (Kahle and Wickham 2013). In the case of the San Andrea isolate, no exact collection coordinates were available, so the location of the San Andrea Chapel in Ravenna, in Italy's Po Valley, the region where most other samples were collected from, was chosen.

### Whole-genome and mChr Sequencing and Genome Assembly

Whole-genome sequencing and assembly of nine Italian blast fungus isolates, including AG006, is described in Win et al. (2020). Briefly, these isolates were sequenced using the PromethION sequencing platform (Oxford Nanopore Technologies, Oxford, UK) and assembled into contigs using Canu (Koren et al. 2017). Assemblies were then polished with Illumina short reads using Pilon (Walker et al. 2014) and Racon (Vaser et al. 2017) and their completeness assessed using BUSCO (Simão et al. 2015), with a 97.7% to 98.8% completeness score taking the ascomycota_odb10 database as input (Win et al. 2020). MCIS of these isolates was performed as described in (Langner et al. 2019, 2021). In short, mChr were separated from core chromosomes using CHEF-based gel electrophoresis. DNA was eluted from gel plugs and sequencing libraries were prepared using a modified version (custom barcodes) of the Nextera Flex library preparation kit (Illumina). Sequencing of mini-chromosomal DNA libraries was carried out on a NextSeq500 system (Illumina).

For whole-genome sequencing and de novo assembly generation of the Br62 isolate, high molecular weight DNA was extracted following (Jones et al. 2021a). Sequencing runs were then performed by Future Genomics Technologies (Leiden, The Netherlands) using the PromethION sequencing platform (Oxford Nanopore Technologies, Oxford, UK). Long reads were assembled into contigs and corrected using Flye (v2.9-b17680) (Kolmogorov et al. 2019) and polished with long reads using Medaka (v1.7.2) (https://github.com/nanoporetech/medaka), and using Illumina short reads (San Diego, USA) through two consecutive iterations of Pilon (v1.23) (Walker et al. 2014). The resulting assembly was of high quality and contiguity, with a BUSCO (Simão et al. 2015) completeness score of 97.4% using the ascomycota_odb10 database and resulting in 10 contigs (supplementary table S10, Supplementary Material online).

### Identification of mChr in Whole-genome Assemblies

MCIS read quality was assessed using fastQC (Andrews 2010). Low quality and adapter sequences were removed using trimmomatic (Bolger et al. 2014). mChr reads were mapped to whole-genome assemblies of each strain using BWA-mem (Li 2013) with default parameters. Reads with multiple mappings (mapping quality = 0) and secondary alignments were removed using samtools (Danecek et al. 2021). MCIS read coverage was calculated in 1 kb sliding windows with a step size of 500 bp using bedtools (Quinlan and Hall 2010). The depth of unambiguously mapping reads was plotted using the R package circlize (Gu et al. 2014). To estimate the repeat content across core and mChr in the nine Italian rice blast isolates, we annotated these using RepeatMasker (http://www.repeatmasker.org/). The input repeat library consisted of the RepBase repeat library for fungi (https://www.girinst.org/repbase/), and repeat libraries from (Chiapello et al. 2015; Peng et al. 2019). For <2Mb contigs repeat content was plotted across 100 kb sliding windows and a step size of 50 kb, while for >2Mb contigs 10 kb windows with a 5 kb step size were chosen.

### Whole-genome and mChr Alignments and Telomere Identification

Whole genome and contig-specific alignments between *M. oryzae* isolates were generated using the nucmer function of MUMMER4 (Marçais et al. 2018). Alignments of a minimum length of 10 kb (–I 10,000) and >80% percent identity (–i 80) were chosen to retrieve contiguous alignments in all pairwise comparisons performed. Alignment coordinates were extracted and whole genome alignments were plotted using the circlize package (Gu et al. 2014). Alignments between individual contigs were visualized with the karyoploteR package (Gel and Serra 2017). We visually inspected mChr contigs for the presence of the canonical telomeric repeats (CCCTAA/TTAGGG)n (Cervenak et al. 2021).

### Genetic Analysis of Blast Fungus Isolates: Mapping and Variant Calling

Illumina short reads of 413 *M. oryzae* and *M. grisea* isolates infecting different host plants (supplementary table S5, Supplementary Material online) were trimmed using AdapterRemoval (v2.3.1) (Schubert et al. 2016) and then mapped to the AG006 reference genome (Win et al. 2020) using bwa-mem (v0.7.17) (Li 2013) with default parameters. Variant identification was performed using GATK (v4.1.4.0) (McKenna et al. 2010). High-quality SNPs were filtered based on the Quality-by-Depth (QD) parameter using GATK's VariantFiltration. Only biallelic SNPs within one standard deviation of the median value of QD scores across all SNPs were kept (Latorre et al. 2022a). To study the phylogenetic relationship between isolates belonging to the rice blast fungus lineage, we subsetted 274 isolates belonging to this lineage (isolates BF5 and BTAr-A1 were removed due to them being outliers in the rice blast fungus phylogeny) and kept informative SNPs with no missing data using VCFtools (v0.1.14). From this dataset, we created a NeighborNet using Splitstree (Huson and Bryant 2006) with concatenated SNPs, resulting in a multi-SNP alignment as input. We repeated this process for members of the *Oryza* clonal lineage II only, and constructed a Maximum-Likelihood (ML) tree using MEGA (v10.2.4) (Kumar et al. 2018), with 100 bootstraps (see data availability). We repeated the same process for the analysis of all 413 isolates shown in Fig. 1 (supplementary table S6, Supplementary Material online). Here, two isolates were removed due to the high amount of missing sites (FR13 and 98 to 06). Based on these SNPs, we created a NJ tree using MEGA (v10.2.4) (Kumar et al. 2018), with 100 bootstraps (see data availability). Isolates deemed as mChrA carriers were highlighted using iTol (Letunic and Bork 2021). To assess for potential discordance in genetic clustering of the core genome and mChrA, we subsetted isolates carrying mChrA (n = 126). For both the core genome and mChrA, only SNPs with a maximum of 10% missing data were kept (−max-missing 0.9) using VCFtools (v0.1.11). NJ trees were constructed using IQtree (v2.03) using fast mode (see data availability). SNP-based PCAs were estimated using the –pca function of PLINK2 (Chang et al. 2015). These were visualized using the R package ggplot2 (v3.4.4, see data availability) (Wickham 2009). To determine the likelihood of the observed genetic discordance being observed by chance, 100 random 1.2Mb regions across the core genome in these 126 isolates were subsetted using a custom python script (see data availability), and NJ trees were computed using IQTree (v2.03) with the fast mode. The number of times each lineage was monophyletic was estimated using a provided custom python script (see data availability).

### mChrC and mChrA Breadth of Coverage Calculations and mChrA-carrier Assignment

To investigate the distribution of the mChrC and mChrA sequence across 413 *M. oryzae* and *M. grisea* isolates, we first calculated the genome-wide breadth of coverage, defined as the percentage of sequence covered by one or more reads from a particular isolate isolate which mapped to the AG006 reference (supplementary table S6, Supplementary Material online). To do this, we estimated breadth of coverage per contig using samtools depth (v1.19) (Danecek et al. 2021), and then created a weighted average taking into account contig length. We assessed breadth of coverage for mChrC (AG006_Contig03) and mChrA (AG006_Contig10) across all isolates and then normalized these values by the isolate's genome-wide breadth of coverage value (supplementary table S6, Supplementary Material online). To determine whether an isolate carried the mChrA sequence or not, we performed clustering using a Gaussian mixture model (GMM) and estimated the Bayesian Information Criterion (BIC) value for 1 to 10 clusters using the R-package mclust (v.6.0.0, see data availability) (Scrucca et al. 2023). Using this same package, we also estimated the uncertainty index for mChrA presence (n = 126) or absence (n = 287) assignment for each isolate (supplementary table S9, Supplementary Material online). We compared the distributions of collection dates between isolates with and without presence of mChrA. We performed a two-sample Wilcoxon test which yielded no difference between the datasets (*P*-value = 0.6877) (supplementary fig. S7, Supplementary Material online). Furthermore, we conducted a logistic regression analysis to assess the probability of mChrA presence or absence based on the collection date. The regression coefficient was not statistically significant (*P*-value = 0.814), indicating that the collection date does not affect the presence of mChrA.

### Ancestry Estimation Using ADMIXTURE

To perform ancestry estimation of isolates carrying the mChrA sequence belonging to different lineages we used ADMIXTURE (v.1.3.0) (Alexander and Lange 2011). Of the 126 isolates carrying mChrA-like sequences, we chose the 62 representative isolates with low amounts of missing genotype information for the mChrA region (supplementary table S5, Supplementary Material online). We chose likely ancestral population numbers (k) based on the estimation of cross-validation (cv) errors within the lower cv range (k = 3 to 10). We performed this analysis both for the core genome, and for mChrA only.

### Identification of mChrA in *Eleusine* Isolate Br62 and mChrA Loss Br62-

*M. oryzae* isolate Br62, belonging to the *Eleusine* lineage, initially carried a single mChr identical in size to mChrA (1.2Mb). To confirm the identity of this mChr, we subcultured Br62 twice via serial passage on Complete Growth Medium (CM), resulting in the loss of mChr as confirmed through CHEF gel electrophoresis. We then sequenced the genome of Br62 without the 1.2Mb mChr (referred to as Br62-) using Illumina short-reads and compared it to the complete Br62 genome sequences. Mapping depth per contig was calculated using the samtools depth function (Danecek et al. 2021). Depths were consistent between Br62 and Br62- except for Contig07, corresponding to mChrA, where Br62- displayed a near-zero read depth. Additionally, repeat content analysis for the Br62 genome, using the same parameters as for the Italian rice blast fungus isolates.

### Annotation and GO Analysis of AG006 and Br62

AG006 and Br62 proteins were translated from gene models predicted from soft-masked genome sequences following the BRAKER2 pipeline (v.2.1.6) (Bruna et al. 2021) using RNA-Seq data (Yan et al. 2023) and the *M. oryzae* 70–15 reference proteome (GenBank Accession Nr: PRJNA13840) as evidence for training. Briefly, genome sequences were softmasked with RepeatMasker (v.4.0.9) (A.F.A. Smit, R. Hubley & P. Green RepeatMasker at http://repeatmasker.org). RNA-seq reads were mapped to chromosomal sequences using HISAT2 (v.2.0.5) (Kim et al. 2019) with default parameters, except for “–max-introlen” set to 5000. Alignment files were then converted to coordinate-sorted bam format using samtools (v.1.10) (Danecek et al. 2021). The bam files containing the RNA-seq alignments were used as evidence to run BRAKER2 with the “–fungus” option to generate a set of gene models. Another set of gene models was predicted using BRAKER2 with the “–epmode –fungus” options and 70–15 proteome as evidence. The two sets of gene models were reconciled into a single set using TSEBRA (Gabriel et al. 2021) with parameters: P 0.1, E 8, C 10, M 1, intron_support 0, stasto_support 1, e_1 0.1, e_2 0.5, e_3 25, e_4 10. This BRAKER2 annotation was complemented by the alignment of two effector datasets (Petit-Houdenot et al. 2020; Yan et al. 2023) to the AG006 and Br62 genomes using miniprot (v0.13-r248) with the options “-G 3k -p 0.3 –outs = 0.5 –gff” (Li 2023). We then extracted the coding sequences (CDSs) in nucleotide form from both BRAKER2 and miniprot GFF files using gffread (v0.12.7) (Pertea and Pertea 2020). Based on the extracted sequences, we filtered out the gene models that lacked complete codons, contained a premature stop codon within the CDS, did not start with a start codon, or were shorter than 150 bp. BRAKER2 and miniprot annotations were merged using gffread with the options “–sort-alpha –force-exons -M -K”. To plot the distribution of gene models, we used their middle positions. When a locus had multiple alternative transcripts, we used the middle position of the locus region and regarded them as a single gene. If any of the alternative transcripts was derived from miniprot or predicted as a putative secreted protein, that locus was regarded as coding for a putative secreted protein. We used the “cut’ function from the Python library “pandas’ (v2.2.1) (McKinney 2011) to count how many genes are in each window. To plot the distribution of repetitive sequences, we counted how many soft-masked bases are in each window.

For both the AG006 and Br62 isolates, we obtained the proteome FASTA files from GFF files using gffread (Pertea and Pertea 2020). We annotated both proteomes using InterProScan (v5.65 to 97.0) with the options “-f gff3 -dp –goterms” (Jones et al. 2014). We then focused on the gene annotations on mChrA in each isolate. We regarded alternative transcripts identified by BRAKER as belonging to the same gene and removed duplicated GO terms from each gene. The resulting GO terms were extracted and visualized using the REVIGO treemap function (Supek et al. 2011). The size of each square represents the -log10(*P* value) of the corresponding GO term (supplementary fig. S12, Supplementary Material online and supplementary tables S15-17).

### Secretome Prediction for AG006 and Br62

AG006 and BR62 secretomes were predicted from their proteomes (Saunders et al. 2012). Briefly, presence of signal peptides in amino acid sequences was predicted using SignalP (v.2.0) (Nielsen and Krogh 1998) with cutoff values of HMM probability of 0.9 or more and a signal cleavage site of 40 or less amino acids. Proteins that were predicted to contain one or more transmembrane domain that did not overlap with the signal peptide, or any mitochondrial transit signals, as predicted by TMHMM (v.2.0) (Krogh et al. 2001) and TargetP (v.2.0) (Armenteros et al. 2019), respectively, were filtered out from the final secretomes. The predicted secretomes were annotated with various features of known effectors using Predector (v.1.2.7) (Jones et al. 2021b).

### Determination of Mating Types

To assign the mating type for each isolate, we created a fasta file containing the nucleotides codifying for the two mating type loci: MAT1-1 (carrying the alpha box motif) (GenBank: BAC65091.1) and MAT1-2 (carrying HMG) (GenBank: BAC65094.1) and used it as a reference genome (Latorre et al. 2022b). We used bwa-mem2 (Li 2013; Vasimuddin et al. 2019) to map each isolate to these sequences and used samtools depth (v1.19) (Danecek et al. 2021) to calculate the breadth of coverage for each locus as a proxy for the mating type assignment (supplementary table S2, Supplementary Material online).

### Differentiation Between Horizontal mChrA Transfer From Introgression via Sexual Mating

To differentiate between horizontal mChr transfer or recent sexual mating or incomplete lineage sorting (ILS) between members of the *Eleusine* and *Oryza* lineages carrying the mChrA sequence, the fixation index (F*_ST_*) based on genome-wide SNPs was calculated. Rice blast isolates carrying mChrA (n = 32) were compared to the two *Eleusine* isolates carrying mChrA, Br62 and B51, using only SNPs with no missing data. Weighted F*_ST_* using 5 kb window sizes and 500 bp step sizes (−fst-window-size 5000 –fst-step-size 500) was calculated using VCFtools (v0.1.14). Next, we assessed patterns of allele sharing and calculated *D-*statistics (Green et al. 2010; Durand et al. 2011) using popstats (Skoglund et al. 2015) as well as using the custom python script *Dstat.py* (see data availability). We removed the mChrA sequence from the *Eleusine* and *Oryza* mChrA carriers and set the *M. grisea* isolate Dig41 as an outgroup, resulting in the following 4-taxa configuration: (Dig41, Br62; *Oryza* +mChrA, *Oryza* -mChrA). The selection of the noncarrier samples (–mChrA) was contingent on their phylogenetic proximity to the tested mChrA carrier (+mChrA) isolate (supplementary table S18, Supplementary Material online). In the case of +mChrA isolate AG006, we performed comparisons against 13 different *Oryza* -mChrA isolates, selected throughout along the different clades of the clonal lineage II. As a control, we also tested the 4-taxa configuration: (Dig41, Br62; *Oryza* +mChrA, *Oryza* +mChrA). The tested isolates were selected based on them having phylogenetic proximity. Complementary to this, we included a second control using the 4-taxa configuration: (Dig41, Br62; *Oryza* -mChrA, *Oryza* -mChrA). The testing pair of isolates were chosen contingent on being part of the same genetic subgroup of the rice blast fungus lineage. In all tested configurations, we only compared rice blast fungus isolates belonging to the same subgroup, to avoid potential unequal drift accumulated between members of different clonal lineages from impacting the analysis. For each configuration we calculated the 99% confidence interval. *D* values were estimated for jack-knife blocks 5Mb and 10Mb in length (supplementary table S18, Supplementary Material online).

### Detection Power of Introgression Using *D* Statistics

We conducted simulations to test the detection power of *D*-statistics under the assumption of a single pulse of introgression followed by multiple backcrosses. We measured Patterson's *D* contingent on: (i) the probability of sexual reproduction per generation; (ii) the number of generations; and (iii) linkage disequilibrium. We first selected a starting four-taxa configuration after removing mChrA sequences. We selected a configuration which yields *D* = 0 and where its counts of ABBA and BABA sites are very similar (Fig. 5a): ((FJ12JN-084-3, 658), Eleusine-infecting_Br62), Dig41. We then recreated a simulation (Scenario 1) beginning with an introgression pulse from Br62 into 658 (D ∼ 1; Z-score >> 3), resulting in an F_1_ individual. This F_1_ individual underwent multiple generations of backcrosses with the parental 658 isolate. Sexual reproduction per generation was modeled as a binomial probability (*P*) ranging from 0.01 to 0.09 and 0.1 to 1.0. After each generation, new mutations, drawn from a Poisson distribution with a lambda of 7e-8 (evolutionary rate from (Latorre et al. 2022a))×genome size, were placed randomly across the genome. We logged *D*-statistics after each generation and estimated the number of generations at which D is statistically indistinguishable from 0 (Z-score < 3.0). We reasoned that Scenario 1 is very conservative, as it involves strict backcrosses with the parental isolate population (supplementary fig. S15a, Supplementary Material online). A more realistic scenario would involve random mating, either with the parental population (Scenario 1) or with another isolate from the same offspring. We modeled this as Scenario 2, logging again D-statistics after each generation and estimating the number of generations at which D is statistically indistinguishable from 0 (Z-score < 3.0) (supplementary fig. S15b, Supplementary Material online).

### Directionality of the Horizontal Transfer

We de novo assembled the Br62 genome using SPAdes (Prjibelski et al. 2020) and identified contigs with high confidence that belong to either mChrA (Contig 10 in the AG006 assembly) or the core chromosomes using minimap2 (Li 2018). If a transfer event occurred from the *Eleusine* to the rice blast fungus lineage, we would see a higher number of shared k-mers between the core chromosomal regions and mChrA regions in Br62, compared to fewer shared k-mers between the core chromosomal regions and mChrA regions in AG006. Conversely, if the transfer event occurred from the rice blast fungus lineage to the *Eleusine* blast fungus lineage, there would be more shared k-mers between the core chromosomal regions and mChrA regions in AG006, compared to Br62. We used k-mer-based Mash distances (Ondov et al. 2016) to measure these patterns (supplementary table S19, Supplementary Material online).

### Differentiating Between a Single and Multiple Horizontal mChrA Transfer Events

To differentiate between a single horizontal ancestral gain of mChrA followed by independent losses and independent horizontal mChrA gains, we measured Kimura two-parameter (K2P) distances in contiguous, nonoverlapping 100kb-sized windows between mChrA in all *Oryza* isolates carrying this sequence (n = 32) and *Eleusine* isolate Br62. We also measured K2P distances across 100 nonoverlapping and randomly sampled 100 kb core chromosomal regions between each isolate and Br62 (see data availability). We then assessed the correlation between averages of the two distance distributions. Both the Pearson's correlation coefficient and its *P*-value were estimated. As a control, we compared average K2P distances between two sets of nonoverlapping and randomly sampled core chromosomal regions to Br62 and again calculated Pearson's correlation coefficient and its *P*-value.

### Dating of Horizontal mChrA Transfer Events Across Clonal Rice Blast Fungus Lineages

In order to infer the dating times of horizontal acquisition of the mChrA sequence in the ancestral nodes of the rice blast fungus phylogeny, we performed a Bayesian-based dated phylogeny using whole-genome concatenated SNPs and incorporating the isolate collection dates (supplementary table S6, Supplementary Material online), and using BEAST2 (v2.7.5) (Bouckaert et al. 2014). We selected the Hasegawa-Kishino-Yano (HKY) nucleotide substitution model. The collection years of the blast fungus isolates served as prior information, providing expected units for the estimated evolutionary rate (substitutions/site/year). We utilized a log-normal distribution with a mean in real space set at 7.5E-8, based on previous estimations (Latorre et al. 2022a). To minimize the effect of demographic assumptions, we chose a Coalescent Extended Bayesian Skyline as a tree prior (Drummond et al. 2005). Isolates without a known collection date were removed from this analysis, and only individuals belonging to rice blast clonal lineages were used to rule out recombination. We ran six independent chains, each spanning a length of 20 million iterations using the CIPRES infrastructure (Miller et al. 2010) and retained the posterior distributions of the estimated trees and their date estimations. To ascertain the ancestral states of presence or absence of the mChrA sequence throughout the rice blast fungus phylogeny, we used the inferred mChrA presence/absence information based on breadth of coverage analyses (supplementary table S9, Supplementary Material online). This was done for all rice blast fungus isolates, as well as for the SA05-43 isolate which belongs to the *Setaria* blast fungus lineage, which was set as an outgroup. These values, which were input as discrete states (mChrA-like sequence carrier = yes/no), were parameterized in a “mugration” analysis, which was implemented in Treetime (v.0.9.0) (Sagulenko et al. 2018) ML-tree as input, generated using IQtree (v2.03) (Minh et al. 2020).

### Textual Enhancement

The articulation of text within this manuscript was assisted by the machine learning model ChatGPT-4.

## Supplementary Material

msae164_Supplementary_Data

## Data Availability

The authors confirm that all data underlying the findings are fully available without restriction. Files and code to perform the analyses described and to generate the plots presented are available as Supplementary Files, and in the git repositories: https://github.com/smlatorreo/mChr_Moryzae (Latorre 2024), https://github.com/CristinaBarragan/Barragan2024_mChr_Moryzae (Barragan 2024) and https://github.com/YuSugihara/Barragan_and_Latorre_et_al_2024 (Sugihara 2024). Sequencing reads were deposited in the European Nucleotide Archive (ENA) under study accession number PRJEB66235 (mini-chromosome sequences from Italian rice blast isolates) and PRJEB67435 (Br62- sequencing). In addition, the Br62 whole-genome assembly is available under GenBank accession number PRJEB66723.

## References

[msae164-B1] Ahmad SF , Martins C. The modern view of B chromosomes under the impact of high scale omics analyses. Cells. 2019:8(2):156. 10.3390/cells8020156. 30781835 PMC6406668

[msae164-B2] Akagi Y , Akamatsu H, Otani H, Kodama M. Horizontal chromosome transfer, a mechanism for the evolution and differentiation of a plant-pathogenic fungus. Eukaryot Cell. 2009:8(11):1732–1738. 10.1128/EC.00135-09. 19749175 PMC2772402

[msae164-B3] Alexander DH , Lange K. Enhancements to the ADMIXTURE algorithm for individual ancestry estimation. BMC Bioinformatics. 2011:12(1):246. 10.1186/1471-2105-12-246. 21682921 PMC3146885

[msae164-B4] Andrews S. 2010. FastQC: a quality control tool for high throughput sequence data. [Computer software]. Available online at: http://www.bioinformatics.babraham.ac.uk/projects/fastqc.

[msae164-B5] Armenteros JJA , Salvatore M, Emanuelsson O, Winther O, von Heijne G, Elofsson A, Nielsenp H. Detecting sequence signals in targeting peptides using deep learning. Life Sci Alliance. 2019:2(5):e201900429. 10.26508/lsa.201900429. 31570514 PMC6769257

[msae164-B6] Asuke S , Horie A, Komatsu K, Mori R, Vy TTP, Inoue Y, Jiang Y, Tatematsu Y, Shimizu M, Tosa Y. Loss of PWT7 located on a supernumerary chromosome is associated with parasitic specialization of Pyricularia oryzae on wheat. Mol Plant Microbe Interact. 2023:36(11):716–725. 10.1094/MPMI-06-23-0078-R. 37432132

[msae164-B8] Aylward AJ , Petrus S, Mamerto A, Hartwick NT, Michael TP. PanKmer: k-mer-based and reference-free pangenome analysis. Bioinformatics. 2023:39:10. 10.1093/bioinformatics/btad621. PMC10603592 37846049

[msae164-B9] Badet T , Croll D. The rise and fall of genes: origins and functions of plant pathogen pangenomes. Curr Opin Plant Biol. 2020:56:65–73. 10.1016/j.pbi.2020.04.009. 32480355

[msae164-B10] Balesdent M-H , Fudal I, Ollivier B, Bally P, Grandaubert J, Eber F, Chèvre A-M, Leflon M, Rouxel T. The dispensable chromosome of leptosphaeria maculans shelters an effector gene conferring avirulence towards Brassica rapa. New Phytol. 2013:198(3):887–898. 10.1111/nph.12178. 23406519

[msae164-B11] Barragan C. 2024. Barragan2024_mChr_Moryzae. Github. Available from: https://github.com/CristinaBarragan/Barragan2024_mChr_Moryzae.

[msae164-B12] Barragan AC , Latorre SM, Mock PG, Harant A, Win J, Malmgren A, Burbano HA, Kamoun S, Langner T. Wild grass isolates of Magnaporthe (Syn. Pyricularia) spp. from Germany can cause blast disease on cereal crops. bioRxiv. 2022. [accessed 2022 August 29]. 10.1101/2022.08.29.505667.

[msae164-B13] Barragan AC , Weigel D. Plant NLR diversity: the known unknowns of pan-NLRomes. Plant Cell. 2021:33(4):814–831. 10.1093/plcell/koaa002. 33793812 PMC8226294

[msae164-B15] Barrett RDH , Schluter D. Adaptation from standing genetic variation. Trends Ecol Evol. 2008:23(1):38–44. 10.1016/j.tree.2007.09.008. 18006185

[msae164-B14] Barrett SCH . Understanding plant reproductive diversity. Philos Trans R Soc Lond B Biol Sci. 2010:365(1537):99–109. 10.1098/rstb.2009.0199. 20008389 PMC2842705

[msae164-B16] Bertazzoni S , Williams AH, Jones DA, Syme RA, Tan K-C, Hane JK. Accessories make the outfit: accessory chromosomes and other dispensable DNA regions in plant-pathogenic fungi. Mol Plant Microbe Interact. 2018:31(8):779–788. 10.1094/MPMI-06-17-0135-FI. 29664319

[msae164-B17] Bhadauria V , MacLachlan R, Pozniak C, Cohen-Skalie A, Li L, Halliday J, Banniza S. Genetic map-guided genome assembly reveals a virulence-governing minichromosome in the lentil anthracnose pathogen colletotrichum lentis. New Phytol. 2019:221(1):431–445. 10.1111/nph.15369. 30076781 PMC6668012

[msae164-B18] Bolger AM , Lohse M, Usadel B. Trimmomatic: a flexible trimmer for illumina sequence data. Bioinformatics. 2014:30(15):2114–2120. 10.1093/bioinformatics/btu170. 24695404 PMC4103590

[msae164-B19] Bouckaert R , Heled J, Kühnert D, Vaughan T, Wu C-H, Xie D, Suchard MA, Rambaut A, Drummond AJ. BEAST 2: a software platform for Bayesian evolutionary analysis. PLoS Comput Biol. 2014:10(4):e1003537. 10.1371/journal.pcbi.1003537. 24722319 PMC3985171

[msae164-B20] Bruna T , Hoff KJ, Lomsadze A, Stanke M, Borodovsky M. BRAKER2: automatic eukaryotic genome annotation with GeneMark-EP+ and AUGUSTUS supported by a protein database. NAR Genom Bioinform. 2021:3(1):lqaa108. 10.1093/nargab/lqaa108. 33575650 PMC7787252

[msae164-B21] Cervenak F , Sepsiova R, Nosek J, Tomaska L. Step-by-Step evolution of telomeres: lessons from yeasts. Genome Biol Evol. 2021:13(2):evaa268. 10.1093/gbe/evaa268. 33537752 PMC7857110

[msae164-B22] Chang CC , Chow CC, Tellier LC, Vattikuti S, Purcell SM, Lee JJ. Second-generation PLINK: rising to the challenge of larger and richer datasets. Gigascience. 2015:4(1):7. 10.1186/s13742-015-0047-8. 25722852 PMC4342193

[msae164-B23] Chiapello H , Mallet L, Guérin C, Aguileta G, Amselem J, Kroj T, Ortega-Abboud E, Lebrun M-H, Henrissat B, Gendrault A, et al Deciphering genome content and evolutionary relationships of isolates from the fungus magnaporthe oryzae attacking different host plants. Genome Biol Evol. 2015:7(10):2896–2912. 10.1093/gbe/evv187. 26454013 PMC4684704

[msae164-B24] Chuma I , Isobe C, Hotta Y, Ibaragi K, Futamata N, Kusaba M, Yoshida K, Terauchi R, Fujita Y, Nakayashiki H, et al Multiple translocation of the AVR-pita effector gene among chromosomes of the rice blast fungus magnaporthe oryzae and related species. PLoS Pathog. 2011:7(7):e1002147. 10.1371/journal.ppat.1002147. 21829350 PMC3145791

[msae164-B25] Chung H , Goh J, Han S-S, Roh J-H, Kim Y, Heu S, Shim H-K, Jeong DG, Kang IJ, Yang J-W. Comparative pathogenicity and host ranges of magnaporthe oryzae and related Species. Plant Pathol J. 2020:36(4):305–313. 10.5423/PPJ.FT.04.2020.0068. 32788889 PMC7403518

[msae164-B26] Couch BC , Fudal I, Lebrun M-H, Tharreau D, Valent B, van Kim P, Nottéghem J-L, Kohn LM. Origins of host-specific populations of the blast pathogen magnaporthe oryzae in crop domestication with subsequent expansion of pandemic clones on rice and weeds of rice. Genetics. 2005:170(2):613–630. 10.1534/genetics.105.041780. 15802503 PMC1450392

[msae164-B27] Covert SF . Supernumerary chromosomes in filamentous fungi. Curr Genet. 1998:33(5):311–319. 10.1007/s002940050342. 9618581

[msae164-B28] Crawford MS , Chumley FG, Weaver CG, Valent B. Characterization of the heterokaryotic and vegetative diploid phases of MAGNAPORTHE GRISEA. Genetics. 1986:114(4):1111–1129. 10.1093/genetics/114.4.1111. 17246357 PMC1203031

[msae164-B29] Croll D , McDonald BA. The accessory genome as a cradle for adaptive evolution in pathogens. PLoS Pathog. 2012:8(4):e1002608. 10.1371/journal.ppat.1002608. 22570606 PMC3343108

[msae164-B30] Cruz CD , Valent B. Wheat blast disease: danger on the move. Trop Plant Pathol. 2017:42(3):210–222. 10.1007/s40858-017-0159-z.

[msae164-B31] Danecek P , Bonfield JK, Liddle J, Marshall J, Ohan V, Pollard MO, Whitwham A, Keane T, McCarthy SA, Davies RM, et al Twelve years of SAMtools and BCFtools. Gigascience. 2021:10(2):giab008. 10.1093/gigascience/giab008. 33590861 PMC7931819

[msae164-B32] Dong S , Raffaele S, Kamoun S. The two-speed genomes of filamentous pathogens: waltz with plants. Curr Opin Genet Dev. 2015:35:57–65. 10.1016/j.gde.2015.09.001. 26451981

[msae164-B33] Drenth A , McTaggart AR, Wingfield BD. Fungal clones win the battle, but recombination wins the war. IMA Fungus. 2019:10(1):18. 10.1186/s43008-019-0020-8. 32647622 PMC7325676

[msae164-B34] Drummond AJ , Rambaut A, Shapiro B, Pybus OG. Bayesian coalescent inference of past population dynamics from molecular sequences. Mol Biol Evol. 2005:22(5):1185–1192. 10.1093/molbev/msi103. 15703244

[msae164-B35] Durand EY , Patterson N, Reich D, Slatkin M. Testing for ancient admixture between closely related populations. Mol Biol Evol. 2011:28(8):2239–2252. 10.1093/molbev/msr048. 21325092 PMC3144383

[msae164-B36] Dylus D , Altenhoff A, Majidian S, Sedlazeck FJ, Dessimoz C. Inference of phylogenetic trees directly from raw sequencing reads using Read2Tree. Nat Biotechnol. 2024:42(1):139–147. 10.1038/s41587-023-01753-4. 37081138 PMC10791578

[msae164-B37] Fitzpatrick DA . Horizontal gene transfer in fungi. FEMS Microbiol Lett. 2012:329(1):1–8. 10.1111/j.1574-6968.2011.02465.x. 22112233

[msae164-B38] Gabaldón T . Patterns and impacts of nonvertical evolution in eukaryotes: a paradigm shift. Ann N Y Acad Sci. 2020:1476(1):78–92. 10.1111/nyas.14471. 32860228 PMC7589212

[msae164-B39] Gabriel L , Hoff KJ, Bruna T, Borodovsky M, Stanke M. TSEBRA: transcript selector for BRAKER. BMC Bioinformatics. 2021:22(1):566. 10.1186/s12859-021-04482-0. 34823473 PMC8620231

[msae164-B40] Gel B , Serra E. Karyoploter: an R/Bioconductor package to plot customizable genomes displaying arbitrary data. Bioinformatics. 2017:33(19):3088–3090. 10.1093/bioinformatics/btx346. 28575171 PMC5870550

[msae164-B41] Gladieux P , Condon B, Ravel S, Soanes D, Maciel JLN, Nhani A Jr, Chen L, Terauchi R, Lebrun M-H, Tharreau D, et al Gene flow between divergent cereal- and grass-specific lineages of the rice blast fungus magnaporthe oryzae. mBio. 2018a:9(1):e01219–e01217. 10.1128/mBio.01219-17. 29487238 PMC5829825

[msae164-B42] Gladieux P , Ravel S, Rieux A, Cros-Arteil S, Adreit H, Milazzo J, Thierry M, Fournier E, Terauchi R, Tharreau D. Coexistence of multiple endemic and pandemic lineages of the rice blast Pathogen. mBio. 2018b:9(2):e01806–e01817. 10.1128/mBio.01806-17. 29615506 PMC5885030

[msae164-B43] Green RE , Krause J, Briggs AW, Maricic T, Stenzel U, Kircher M, Patterson N, Li H, Zhai W, Fritz MH-Y, et al A draft sequence of the Neandertal genome. Science. 2010:328(5979):710–722. 10.1126/science.1188021. 20448178 PMC5100745

[msae164-B44] Gu Z , Gu L, Eils R, Schlesner M, Brors B. Circlize implements and enhances circular visualization in R. Bioinformatics. 2014:30(19):2811–2812. 10.1093/bioinformatics/btu393. 24930139

[msae164-B45] Gyawali N , Hao Y, Lin G, Huang J, Bika R, Daza LC, Zheng H, Cruppe G, Caragea D, Cook D, et al Using recurrent neural networks to detect supernumerary chromosomes in fungal strains causing blast diseases. bioRxiv 558148. 10.1101/2023.09.17.558148., 18 September 2023, preprint: not peer reviewed. PMC11333962 39165675

[msae164-B46] Habig M , Grasse AV, Mueller J, Stukenbrock EH, Leitner H, Cremer S. Frequent horizontal chromosome transfer between asexual fungal insect pathogens. Proc Natl Acad Sci U S A. 2023:121(11):e2316284121. 10.1073/pnas.2316284121. PMC10945790 38442176

[msae164-B47] Habig M , Quade J, Stukenbrock EH. Forward genetics approach reveals host genotype-dependent importance of accessory chromosomes in the fungal wheat pathogen zymoseptoria tritici. mBio. 2017:8(6):e01919–e01917. 10.1128/mBio.01919-17. 29184021 PMC5705923

[msae164-B48] Han Y , Liu X, Benny U, Kistler HC, VanEtten HD. Genes determining pathogenicity to pea are clustered on a supernumerary chromosome in the fungal plant pathogen nectria haematococca. Plant J. 2001:25(3):305–314. 10.1046/j.1365-313x.2001.00969.x. 11208022

[msae164-B49] He C , Rusu AG, Poplawski AM, Irwin JA, Manners JM. Transfer of a supernumerary chromosome between vegetatively incompatible biotypes of the fungus colletotrichum gloeosporioides. Genetics. 1998:150(4):1459–1466. 10.1093/genetics/150.4.1459. 9832523 PMC1460434

[msae164-B50] Henry PM , Pincot DDA, Jenner BN, Borrero C, Aviles M, Nam M-H, Epstein L, Knapp SJ, Gordon TR. Horizontal chromosome transfer and independent evolution drive diversification in fusarium oxysporum f. sp. fragariae. New Phytol. 2021:230(1):327–340. 10.1111/nph.17141. 33616938 PMC7986148

[msae164-B51] Huang J , Liu S, Cook DE. Dynamic genomes - mechanisms and consequences of genomic diversity impacting plant-fungal interactions. Physiol Mol Plant Pathol. 2023:125:102006. 10.1016/j.pmpp.2023.102006.

[msae164-B52] Huson DH , Bryant D. Application of phylogenetic networks in evolutionary studies. Mol Biol Evol. 2006:23(2):254–267. 10.1093/molbev/msj030. 16221896

[msae164-B53] Hyon G-S , Nga NTT, Chuma I, Inoue Y, Asano H, Murata N, Kusaba M, Tosa Y. Characterization of interactions between barley and various host-specific subgroups of magnaporthe oryzae and M. grisea. J Gen Plant Pathol. 2012:78(4):237–246. 10.1007/s10327-012-0386-6.

[msae164-B54] Ishikawa FH , Barcelos QL, Alves E, Camargo OA Jr, de Souza EA. Symptoms and prepenetration events associated with the infection of common bean by the anamorph and teleomorph of Glomerella cingulata f.sp. phaseoli. J. Phytopathol. 2010:158(4):270–277. 10.1111/j.1439-0434.2009.01608.x.

[msae164-B55] Islam T , Ansary MWR, Rahman MM. Magnaporthe oryzae and its pathotypes: a potential plant pandemic threat to global food security. In: Scott B, Mesarich C, editors. Plant relationships: fungal-plant interactions. Cham: Springer International Publishing; 2023. p. 425–462.

[msae164-B58] Jones A , Torkel C, Stanley D, Nasim J, Borevitz J, Schwessinger B. High-molecular weight DNA extraction, clean-up and size selection for long-read sequencing. PLoS One. 2021a:16(7):e0253830. 10.1371/journal.pone.0253830. 34264958 PMC8282028

[msae164-B56] Jones P , Binns D, Chang H-Y, Fraser M, Li W, McAnulla C, McWilliam H, Maslen J, Mitchell A, Nuka G, et al InterProScan 5: genome-scale protein function classification. Bioinformatics. 2014:30(9):1236–1240. 10.1093/bioinformatics/btu031. 24451626 PMC3998142

[msae164-B57] Jones DAB , Rozano L, Debler JW, Mancera RL, Moolhuijzen PM, Hane JK. Publisher correction: an automated and combinative method for the predictive ranking of candidate effector proteins of fungal plant pathogens. Sci Rep. 2021b:11(1):24168. 10.1038/s41598-021-03673-2. 34903839 PMC8668936

[msae164-B59] Kahle D , Wickham H. Ggmap: spatial visualization with ggplot2. R J. 2013:5(1):144–161. 10.32614/RJ-2013-014.

[msae164-B60] Kato H , Yamamoto M, Yamaguchi-Ozaki T, Kadouchi H, Iwamoto Y, Nakayashiki H, Tosa Y, Mayama S, Mori N. Pathogenicity, mating ability and DNA restriction fragment length polymorphisms of pyricularia populations isolated from gramineae, bambusideae and Zingiberaceae plants. J. Gen Plant Pathol. 2000:66(1):30–47. 10.1007/PL00012919.

[msae164-B61] Kim D , Paggi JM, Park C, Bennett C, Salzberg SL. Graph-based genome alignment and genotyping with HISAT2 and HISAT-genotype. Nat Biotechnol. 2019:37(8):907–915. 10.1038/s41587-019-0201-4. 31375807 PMC7605509

[msae164-B62] Kistler CH . Mutants ofNectria haematococcaCreated by a site-directed chromosome breakage are greatly reduced in virulence toward pea. Mol Plant Microbe Interact. 1996:9(9):804. 10.1094/MPMI-9-0804.

[msae164-B63] Kobayashi N , Dang TA, Kieu PTM, Gómez Luciano LB, Van Ba V, Izumitsu K, Shimizu M, Ikeda K-I, Li W-H, Nakayashiki H. Horizontally transferred DNA in the genome of the fungus Pyricularia oryzae is associated with repressive histone modifications. Mol Biol Evol. 2023:40(9):msad186. 10.1093/molbev/msad186. 37595132 PMC10473863

[msae164-B64] Kolmogorov M , Yuan J, Lin Y, Pevzner PA. Assembly of long, error-prone reads using repeat graphs. Nat Biotechnol. 2019:37(5):540–546. 10.1038/s41587-019-0072-8. 30936562

[msae164-B65] Koren S , Walenz BP, Berlin K, Miller JR, Bergman NH, Phillippy AM. Canu: scalable and accurate long-read assembly via adaptive k-mer weighting and repeat separation. Genome Res. 2017:27(5):722–736. 10.1101/gr.215087.116. 28298431 PMC5411767

[msae164-B66] Krogh A , Larsson B, von Heijne G, Sonnhammer EL. Predicting transmembrane protein topology with a hidden markov model: application to complete genomes. J Mol Biol. 2001:305(3):567–580. 10.1006/jmbi.2000.4315. 11152613

[msae164-B67] Kumar S , Stecher G, Li M, Knyaz C, Tamura K. MEGA x: molecular evolutionary genetics analysis across computing platforms. Mol Biol Evol. 2018:35(6):1547–1549. 10.1093/molbev/msy096. 29722887 PMC5967553

[msae164-B68] Kusaba M , Mochida T, Naridomi T, Fujita Y, Chuma I, Tosa Y. Loss of a 1.6 Mb chromosome in pyricularia oryzae harboring two alleles of AvrPik leads to acquisition of virulence to rice cultivars containing resistance alleles at the Pik locus. Curr Genet. 2014:60(4):315–325. 10.1007/s00294-014-0437-y. 25056242

[msae164-B69] Langner T , Harant A, Gomez-Luciano LB, Shrestha RK, Malmgren A, Latorre SM, Burbano HA, Win J, Kamoun S. Genomic rearrangements generate hypervariable mini-chromosomes in host-specific isolates of the blast fungus. PLoS Genet. 2021:17(2):e1009386. 10.1371/journal.pgen.1009386. 33591993 PMC7909708

[msae164-B70] Langner T , Harant A, Kamoun S. Isolation of supernumerary mini-chromosomes from fungi for enrichment sequencing. bioRxiv. 2019. [accessed 2019 December 27]. https://doi.org/10.1750.

[msae164-B71] Latorre SM. smlatorreo/mChr_Moryzae: mChr_Moryzae. 2024. [accessed 2024 February 7]. Available from: https://zenodo.org/doi/10.5281/zenodo.10628812.

[msae164-B72] Latorre SM , Langner T, Malmgren A, Win J, Kamoun S, Burbano HA. SNP calling parameters have minimal impact on population structure and divergence time estimates for the rice blast fungus. bioRxiv 482794. 10.1101/2022.03.06.482794, 7 March 2022a, preprint: not peer reviewed.

[msae164-B73] Latorre SM , Reyes-Avila CS, Malmgren A, Win J, Kamoun S, Burbano HA. Differential loss of effector genes in three recently expanded pandemic clonal lineages of the rice blast fungus. BMC Biol. 2020:18(1):88. 10.1186/s12915-020-00818-z. 32677941 PMC7364606

[msae164-B74] Latorre SM , Were VM, Foster AJ, Langner T, Malmgren A, Harant A, Asuke S, Reyes-Avila S, Gupta DR, Jensen C, et al Genomic surveillance uncovers a pandemic clonal lineage of the wheat blast fungus. PLoS Biol. 2023:21(4):e3002052. 10.1371/journal.pbio.3002052. 37040332 PMC10089362

[msae164-B7] Latorre SM , Were VM, Langer T, Foster AJ, Win J, Kamoun S, Talbot NJ, Burbano HA. 2022b. A curated set of mating-type assignment for the blast fungus (Magnaporthales). Available from: https://zenodo.org/record/6369833

[msae164-B75] Letunic I , Bork P. Interactive tree of life (iTOL) v5: an online tool for phylogenetic tree display and annotation. Nucleic Acids Res. 2021:49(W1):W293–W296. 10.1093/nar/gkab301. 33885785 PMC8265157

[msae164-B76] Li H. Aligning sequence reads, clone sequences and assembly contigs with BWA-MEM. arXiv 1303.3997. 10.48550/arXiv.1303.3997, 26 May 2013, preprint: not peer reviewed.

[msae164-B77] Li H . Minimap2: pairwise alignment for nucleotide sequences. Bioinformatics. 2018:34(18):3094–3100. 10.1093/bioinformatics/bty191. 29750242 PMC6137996

[msae164-B78] Li H . Protein-to-genome alignment with miniprot. Bioinformatics. 2023:39(1):btad014. 10.1093/bioinformatics/btad014. 36648328 PMC9869432

[msae164-B79] Liu S , Lin G, Ramachandran SR, Cruppe G, Cook D, Pedley KF, Valent B. Rapid mini-chromosome divergence among fungal isolates causing wheat blast outbreaks in Bangladesh and Zambia. bioRxiv 496690. 10.1101/2022.06.18.496690, 19 June 2022, preprint: not peer reviewed. 37984076

[msae164-B80] Ma L-J , van der Does HC, Borkovich KA, Coleman JJ, Daboussi M-J, Di Pietro A, Dufresne M, Freitag M, Grabherr M, Henrissat B, et al Comparative genomics reveals mobile pathogenicity chromosomes in Fusarium. Nature. 2010:464(7287):367–373. 10.1038/nature08850. 20237561 PMC3048781

[msae164-B81] Marçais G , Delcher AL, Phillippy AM, Coston R, Salzberg SL, Zimin A. MUMmer4: a fast and versatile genome alignment system. PLoS Comput Biol. 2018:14(1):e1005944. 10.1371/journal.pcbi.1005944. 29373581 PMC5802927

[msae164-B82] Masel AM , He C, Poplawski AM, Irwin JAG, Manners JM. Molecular evidence for chromosome transfer between biotypes of Colletotrichum gloeosporioides. Mol Plant Microbe Interact. 1996:9(5):339–348. 10.1094/MPMI-9-0339.

[msae164-B83] McCarthy CGP , Fitzpatrick DA. Pan-genome analyses of model fungal species. Microb Genom. 2019:5(2):e000243. 10.1099/mgen.0.000243. 30714895 PMC6421352

[msae164-B84] McKenna A , Hanna M, Banks E, Sivachenko A, Cibulskis K, Kernytsky A, Garimella K, Altshuler D, Gabriel S, Daly M, et al The genome analysis toolkit: a MapReduce framework for analyzing next-generation DNA sequencing data. Genome Res. 2010:20(9):1297–1303. 10.1101/gr.107524.110. 20644199 PMC2928508

[msae164-B85] McKinney W . Pandas: a foundational Python library for data analysis and statistics. Python for high performance and scientific computing [Internet]. 2011:14(9):1–9. Available from: https://www.dlr.de/sc/portaldata/15/resources/dokumente/pyhpc2011/submissions/pyhpc2011_submission_9.pdf.

[msae164-B86] Mehrabi R , Bahkali AH, Abd-Elsalam KA, Moslem M, Ben M’barek S, Gohari AM, Jashni MK, Stergiopoulos I, Kema GHJ, de Wit PJGM. Horizontal gene and chromosome transfer in plant pathogenic fungi affecting host range. FEMS Microbiol Rev. 2011:35(3):542–554. 10.1111/j.1574-6976.2010.00263.x. 21223323

[msae164-B87] Miao VP , Covert SF, VanEtten HD. A fungal gene for antibiotic resistance on a dispensable (“B”) chromosome. Science. 1991:254(5039):1773–1776. 10.1126/science.1763326. 1763326

[msae164-B88] Miller MA , Pfeiffer W, Schwartz T. Creating the CIPRES science gateway for inference of large phylogenetic trees. In: 2010 Gateway computing environments workshop (GCE). New Orleans, USA: IEEE. p. 1–8.

[msae164-B89] Minh BQ , Schmidt HA, Chernomor O, Schrempf D, Woodhams MD, von Haeseler A, Lanfear R. IQ-TREE 2: new models and efficient methods for phylogenetic inference in the genomic era. Mol Biol Evol. 2020:37(5):1530–1534. 10.1093/molbev/msaa015. 32011700 PMC7182206

[msae164-B90] Mohanta TK , Bae H. The diversity of fungal genome. Biol Proced Online. 2015:17(1):8. 10.1186/s12575-015-0020-z. 25866485 PMC4392786

[msae164-B91] Möller M , Stukenbrock EH. Evolution and genome architecture in fungal plant pathogens. Nat Rev Microbiol. 2017:15(12):756–771. 10.1038/nrmicro.2017.76. 29123226

[msae164-B92] Monsur MA , Kusaba M. Study on parasexual recombination between pyricularia oryzae and pyricularia grisea. Agric Sci China. 2018:9:317–339. 10.4236/as.2018.93023.

[msae164-B93] Moolhuijzen PM , See PT, Shi G, Powell HR, Cockram J, Jørgensen LN, Benslimane H, Strelkov SE, Turner J, Liu Z, et al A global pangenome for the wheat fungal pathogen Pyrenophora tritici-repentis and prediction of effector protein structural homology. Microb Genom. 2022:8(10):mgen000872. 10.1099/mgen.0.000872. 36214662 PMC9676058

[msae164-B94] Nei M . The new mutation theory of phenotypic evolution. Proc Natl Acad Sci U S A. 2007:104(30):12235–12242. 10.1073/pnas.0703349104. 17640887 PMC1941456

[msae164-B95] Nielsen H , Krogh A. Prediction of signal peptides and signal anchors by a hidden markov model. Proc Int Conf Intell Syst Mol Biol. 1998:6:122–130. 9783217

[msae164-B96] Nieuwenhuis BPS , James TY. The frequency of sex in fungi. Philos Trans R Soc Lond B Biol Sci. 2016:371(1706):20150540. 10.1098/rstb.2015.0540. 27619703 PMC5031624

[msae164-B97] Noguchi MT , Yasuda N, Fujita Y. Evidence of genetic exchange by parasexual recombination and genetic analysis of pathogenicity and mating type of parasexual recombinants in rice blast fungus, magnaporthe oryzae. Phytopathology. 2006:96(7):746–750. 10.1094/PHYTO-96-0746. 18943148

[msae164-B98] Oggenfuss U , Feurtey A, Reyes-Avila CS, Gluck-Thaler E, Puccetti G, Glad HM, Abraham LN, Stalder L, Tralamazza SM, González-Sáyer SM, et al Genome evolution in fungal plant pathogens: from populations to kingdom-wide dynamics. In: Pöggeler S, James T, editors. Evolution of fungi and fungal-like organisms. Cham: Springer International Publishing; 2023. p. 103–121.

[msae164-B99] Ondov BD , Treangen TJ, Melsted P, Mallonee AB, Bergman NH, Koren S, Phillippy AM. Mash: fast genome and metagenome distance estimation using MinHash. Genome Biol. 2016:17(1):132. 10.1186/s13059-016-0997-x. 27323842 PMC4915045

[msae164-B100] Orbach MJ , Chumley FG, Valent B. Electrophoretic karyotypes of Magnaporthe grisea pathogens of diverse grasses. MPMI-Molecular Plant Microbe Interactions. 1996:9(4):261–271. 10.1094/MPMI-9-0261.

[msae164-B101] Peng Z , Oliveira-Garcia E, Lin G, Hu Y, Dalby M, Migeon P, Tang H, Farman M, Cook D, White FF, et al Effector gene reshuffling involves dispensable mini-chromosomes in the wheat blast fungus. PLoS Genet. 2019:15(9):e1008272. 10.1371/journal.pgen.1008272. 31513573 PMC6741851

[msae164-B102] Pertea G , Pertea M. GFF utilities: gffRead and GffCompare. F1000Res. 2020:9(304). 10.12688/f1000research.23297.2. PMC7222033 32489650

[msae164-B103] Petersen C , Sørensen T, Nielsen MR, Sondergaard TE, Sørensen JL, Fitzpatrick DA, Frisvad JC, Nielsen KL. Comparative genomic study of the Penicillium genus elucidates a diverse pangenome and 15 lateral gene transfer events. IMA Fungus. 2023:14(1):3. 10.1186/s43008-023-00108-7. 36726175 PMC9893605

[msae164-B104] Petit-Houdenot Y , Langner T, Harant A, Win J, Kamoun S. A clone resource of magnaporthe oryzae effectors that share sequence and structural similarities across host-specific lineages. Mol Plant Microbe Interact. 2020:33(8):1032–1035. 10.1094/MPMI-03-20-0052-A. 32460610

[msae164-B105] Potgieter L , Feurtey A, Dutheil JY, Stukenbrock EH. On variant discovery in genomes of fungal plant pathogens. Front Microbiol. 2020:11:626. 10.3389/fmicb.2020.00626. 32373089 PMC7176817

[msae164-B106] Prjibelski A , Antipov D, Meleshko D, Lapidus A, Korobeynikov A. Using SPAdes De Novo assembler. Curr Protoc Bioinformatics. 2020:70(1):e102. 10.1002/cpbi.102. 32559359

[msae164-B107] Quinlan AR , Hall IM. BEDTools: a flexible suite of utilities for comparing genomic features. Bioinformatics. 2010:26(6):841–842. 10.1093/bioinformatics/btq033. 20110278 PMC2832824

[msae164-B108] Rahman MT , Sobur MA, Islam MS, Ievy S, Hossain MJ, El Zowalaty ME, Rahman AT, Ashour HM. Zoonotic diseases: etiology, impact, and control. Microorganisms. 2020:8(9):1405. 10.3390/microorganisms8091405. 32932606 PMC7563794

[msae164-B109] Rahnama M , Novikova O, Starnes JH, Zhang S, Chen L, Farman ML. Transposon-mediated telomere destabilization: a driver of genome evolution in the blast fungus. Nucleic Acids Res. 2020:48(13):7197–7217. 10.1093/nar/gkaa287. 32558886 PMC7367193

[msae164-B110] Roca MG , Arlt J, Jeffree CE, Read ND. Cell biology of conidial anastomosis tubes in Neurospora crassa. Eukaryot Cell. 2005:4(5):911–919. 10.1128/EC.4.5.911-919.2005. 15879525 PMC1140100

[msae164-B111] Roca MG , Davide LC, Mendes-Costa MC, Wheals A. Conidial anastomosis tubes in Colletotrichum. Fungal Genet Biol. 2003:40(2):138–145. 10.1016/S1087-1845(03)00088-4. 14516766

[msae164-B112] Roper M , Ellison C, Taylor JW, Glass NL. Nuclear and genome dynamics in multinucleate ascomycete fungi. Curr Biol. 2011:21(18):R786–R793. 10.1016/j.cub.2011.06.042. 21959169 PMC3184236

[msae164-B113] Sagulenko P , Puller V, Neher RA. TreeTime: maximum-likelihood phylodynamic analysis. Virus Evol. 2018:4(1):vex042. 10.1093/ve/vex042. 29340210 PMC5758920

[msae164-B114] Sahu N , Indic B, Wong-Bajracharya J, Merényi Z, Ke H-M, Ahrendt S, Monk T-L, Kocsubé S, Drula E, Lipzen A, et al Vertical and horizontal gene transfer shaped plant colonization and biomass degradation in the fungal genus Armillaria. Nat Microbiol. 2023:8(9):1668–1681. 10.1038/s41564-023-01448-1. 37550506 PMC7615209

[msae164-B115] Saleh D , Xu P, Shen Y, Li C, Adreit H, Milazzo J, Ravigné V, Bazin E, Nottéghem J-L, Fournier E, et al Sex at the origin: an Asian population of the rice blast fungus Magnaporthe oryzae reproduces sexually. Mol Ecol. 2012:21(6):1330–1344. 10.1111/j.1365-294X.2012.05469.x. 22313491

[msae164-B116] Saunders DGO , Win J, Cano LM, Szabo LJ, Kamoun S, Raffaele S. Using hierarchical clustering of secreted protein families to classify and rank candidate effectors of rust fungi. PLoS One. 2012:7(1):e29847. 10.1371/journal.pone.0029847. 22238666 PMC3253089

[msae164-B117] Schubert M , Lindgreen S, Orlando L. AdapterRemoval v2: rapid adapter trimming, identification, and read merging. BMC Res Notes. 2016:9(1):88. 10.1186/s13104-016-1900-2. 26868221 PMC4751634

[msae164-B118] Scrucca L , Fraley C, Murphy TB, Raftery AE. Model-based clustering, classification, and density estimation using mclust in R. 1st ed. New York, USA: Chapman and Hall/CRC; 2023.

[msae164-B119] Seidl MF , Thomma BPHJ. Sex or no sex: evolutionary adaptation occurs regardless. Bioessays. 2014:36(4):335–345. 10.1002/bies.201300155. 24531982 PMC4158867

[msae164-B120] Simão FA , Waterhouse RM, Ioannidis P, Kriventseva EV, Zdobnov EM. BUSCO: assessing genome assembly and annotation completeness with single-copy orthologs. Bioinformatics. 2015:31(19):3210–3212. 10.1093/bioinformatics/btv351. 26059717

[msae164-B121] Skoglund P , Mallick S, Bortolini MC, Chennagiri N, Hünemeier T, Petzl-Erler ML, Salzano FM, Patterson N, Reich D. Genetic evidence for two founding populations of the Americas. Nature. 2015:525(7567):104–108. 10.1038/nature14895. 26196601 PMC4982469

[msae164-B122] Soanes D , Richards TA. Horizontal gene transfer in eukaryotic plant pathogens. Annu Rev Phytopathol. 2014:52(1):583–614. 10.1146/annurev-phyto-102313-050127. 25090479

[msae164-B123] Strom NB , Bushley KE. Two genomes are better than one: history, genetics, and biotechnological applications of fungal heterokaryons. Fungal Biol Biotechnol. 2016:3(1):4. 10.1186/s40694-016-0022-x. 28955463 PMC5611628

[msae164-B124] Stukenbrock EH . Evolution, selection and isolation: a genomic view of speciation in fungal plant pathogens. New Phytol. 2013:199(4):895–907. 10.1111/nph.12374. 23782262

[msae164-B125] Stukenbrock EH . The role of hybridization in the evolution and emergence of new fungal plant pathogens. Phytopathology. 2016:106(2):104–112. 10.1094/PHYTO-08-15-0184-RVW. 26824768

[msae164-B146] Sugihara Y. 2024. YuSugihara/Barragan_and_Latorre_et_al_2024. https://zenodo.org/records/11111761

[msae164-B126] Sun D . Pull in and push out: mechanisms of horizontal gene transfer in Bacteria. Front Microbiol. 2018:9:2154. 10.3389/fmicb.2018.02154. 30237794 PMC6135910

[msae164-B127] Supek F , Bošnjak M, Škunca N, Šmuc T. REVIGO summarizes and visualizes long lists of gene ontology terms. PLoS One. 2011:6(7):e21800. 10.1371/journal.pone.0021800. 21789182 PMC3138752

[msae164-B128] Takeuchi N , Kaneko K, Koonin EV. Horizontal gene transfer can rescue prokaryotes from Muller's ratchet: benefit of DNA from dead cells and population subdivision. G3 (Bethesda). 2014:4(2):325–339. 10.1534/g3.113.009845. 24347631 PMC3931566

[msae164-B129] Talbot NJ , Salch YP, Ma M, Hamer JE. Karyotypic variation within clonal lineages of the rice blast fungus, magnaporthe grisea. Appl Environ Microbiol. 1993:59(2):585–593. 10.1128/aem.59.2.585-593.1993. 16348876 PMC202148

[msae164-B130] Thierry M , Charriat F, Milazzo J, Adreit H, Ravel S, Cros-Arteil S, Borron S, Sella V, Kroj T, Ioos R, et al Maintenance of divergent lineages of the rice blast fungus pyricularia oryzae through niche separation, loss of sex and post-mating genetic incompatibilities. PLoS Pathog. 2022:18(7):e1010687. 10.1371/journal.ppat.1010687. 35877779 PMC9352207

[msae164-B131] Torres DE , Oggenfuss U, Croll D, Seidl MF. Genome evolution in fungal plant pathogens: looking beyond the two-speed genome model. Fungal Biol Rev. 2020:34(3):136–143. 10.1016/j.fbr.2020.07.001.

[msae164-B132] Tsujimoto NM . Parasexual recombination in magnaporthe oryzae. Jpn Agric Res Q. 2011:45(1):39–45. 10.6090/jarq.45.39.

[msae164-B133] van Dam P , Fokkens L, Ayukawa Y, van der Gragt M, ter Horst A, Brankovics B, Houterman PM, Arie T, Rep M. A mobile pathogenicity chromosome in fusarium oxysporum for infection of multiple cucurbit species. Sci Rep. 2017:7(1):1–15. 10.1038/s41598-017-07995-y. 28831051 PMC5567276

[msae164-B134] Vangalis V , Knop M, Typas MA, Papaioannou IA. Establishment of conidial fusion in the asexual fungus Verticillium dahliae as a useful system for the study of non-sexual genetic interactions. Curr Genet. 2021:67(3):471–485. 10.1007/s00294-021-01157-4. 33582843 PMC8139932

[msae164-B135] Van Valen L . A new evolutionary law. Evol Theory. 1973:1:31–49.

[msae164-B136] van Westerhoven A , Aguilera-Galvez C, Nakasato-Tagami G, Shi-Kunne X, Dijkstra J, de la Parte EM, Carero EC, Meijer H, Feurtey A, Maryani N, et al Segmental duplications drive the evolution of accessory regions in a Major crop pathogen. bioRxiv 544053. 10.1101/2023.06.07.544053, 7 June 2023, preprint: not peer reviewed. 38402521

[msae164-B137] Vaser R , Sović I, Nagarajan N, Šikić M. Fast and accurate de novo genome assembly from long uncorrected reads. Genome Res. 2017:27(5):737–746. 10.1101/gr.214270.116. 28100585 PMC5411768

[msae164-B138] Vasimuddin M , Misra S, Li H, Aluru S. Efficient architecture-aware acceleration of BWA-MEM for multicore systems. In: 2019 IEEE international parallel and distributed processing symposium (IPDPS). Rio de Janeiro, Brazil: IEEE. p. 314–324.

[msae164-B139] Vlaardingerbroek I , Beerens B, Rose L, Fokkens L, Cornelissen BJC, Rep M. Exchange of core chromosomes and horizontal transfer of lineage-specific chromosomes in fusarium oxysporum. Environ Microbiol. 2016:18(11):3702–3713. 10.1111/1462-2920.13281. 26941045

[msae164-B140] Walker BJ , Abeel T, Shea T, Priest M, Abouelliel A, Sakthikumar S, Cuomo CA, Zeng Q, Wortman J, Young SK, et al Pilon: an integrated tool for comprehensive microbial variant detection and genome assembly improvement. PLoS One. 2014:9(11):e112963. 10.1371/journal.pone.0112963. 25409509 PMC4237348

[msae164-B141] Wickham H . Ggplot2: elegant graphics for data analysis. New York City, USA: Springer Science & Business Media; 2009.

[msae164-B142] Win J , Harant A, Malmgren A, Langner T, Shrestha R-K, Latorre SM, Were V, Talbot NJ, Burbano HA, Picco AM, et al Large scale genome assemblies of Magnaporthe oryzae rice isolates from Italy. 2020. [accessed 2020 December 16]. Available from: https://zenodo.org/record/4326823.

[msae164-B143] Wright S . The genetical structure of populations. Ann Eugen. 1951:15(1):323–354. 10.1111/j.1469-1809.1949.tb02451.x. 24540312

[msae164-B144] Yan X , Tang B, Ryder LS, MacLean D, Were VM, Eseola AB, Cruz-Mireles N, Ma W, Foster AJ, Osés-Ruiz M, et al The transcriptional landscape of plant infection by the rice blast fungus magnaporthe oryzae reveals distinct families of temporally co-regulated and structurally conserved effectors. Plant Cell. 2023:35(5):1360–1385. 10.1093/plcell/koad036. 36808541 PMC10118281

[msae164-B145] Younas MU , Wang G, Du H, Zhang Y, Ahmad I, Rajput N, Li M, Feng Z, Hu K, Khan NU, et al Approaches to reduce rice blast disease using knowledge from host resistance and pathogen pathogenicity. Int J Mol Sci. 2023:24(5):4985. 10.3390/ijms24054985. 36902415 PMC10003181

[msae164-B147] Zeigler RS , Scott RP, Leung H, Bordeos AA, Kumar J, Nelson RJ. Evidence of parasexual exchange of DNA in the rice blast fungus challenges its exclusive clonality. Phytopathology. 1997:87(3):284–294. 10.1094/PHYTO.1997.87.3.284. 18945171

[msae164-B148] Zielezinski A , Vinga S, Almeida J, Karlowski WM. Alignment-free sequence comparison: benefits, applications, and tools. Genome Biol. 2017:18(1):186. 10.1186/s13059-017-1319-7. 28974235 PMC5627421

